# Physics of 2D Materials for Developing Smart Devices

**DOI:** 10.1007/s40820-024-01635-7

**Published:** 2025-03-21

**Authors:** Neeraj Goel, Rahul Kumar

**Affiliations:** 1 Department of Electronics and Communication Engineering, Netaji Subhas University of Technology, Dwarka, New Delhi 110078 India; 2 Institute of Infrastructure Technology Research and Management, Ahmedabad, 380026 India

**Keywords:** 2D materials, Heterostructures, Smart devices, Van der Waals, Flexible electronics

## Abstract

Rapid industrialization advancements have grabbed worldwide attention to integrate a very large number of electronic components into a smaller space for performing multifunctional operations. To fulfill the growing computing demand state-of-the-art materials are required for substituting traditional silicon and metal oxide semiconductors frameworks. Two-dimensional (2D) materials have shown their tremendous potential surpassing the limitations of conventional materials for developing smart devices. Despite their ground-breaking progress over the last two decades, systematic studies providing in-depth insights into the exciting physics of 2D materials are still lacking. Therefore, in this review, we discuss the importance of 2D materials in bridging the gap between conventional and advanced technologies due to their distinct statistical and quantum physics. Moreover, the inherent properties of these materials could easily be tailored to meet the specific requirements of smart devices. Hence, we discuss the physics of various 2D materials enabling them to fabricate smart devices. We also shed light on promising opportunities in developing smart devices and identified the formidable challenges that need to be addressed.

## Highlights

- Extensively discussed the physics of various two-dimensional materials enabling them to fabricate smart devices.
- Statistical and quantum physics for understanding the functioning of smart electronic devices with strategies for improving their performance.
- New advancement in device architectures for developing smart devices.

## Graphical abstract

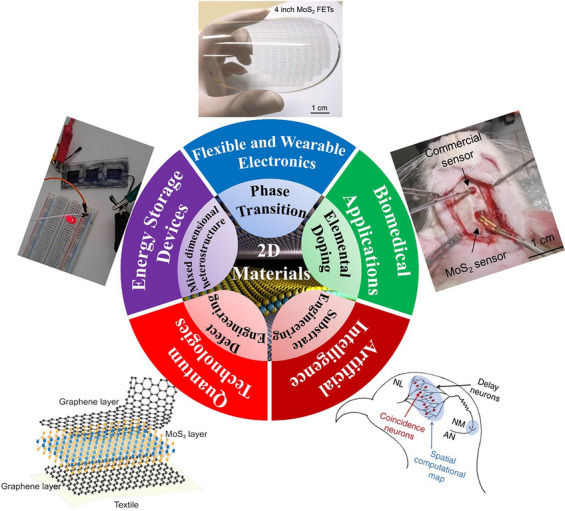

## Introduction

In 1965, Gordon Moore stated that the number of transistors on a chip doubles every two years. Over the last 5 decades, Moore’s law has worked fairly well, driving significant improvement in performance and a sharp decrease in the prices of electronic circuits. However, it starts saturating particularly due to the introduction of multifunctional devices as a very large number of components are required to be embedded into the same system leading to excessive heat generated within the chip [1]. Thus, the scientific community has to look beyond conventional materials to revive Moore’s law for making high-performance and low-dimension smart devices. The atomically thinned layered materials popularly known as two-dimensional (2D) materials showed a high probability of bringing back Moore’s law to life. The properties of these 2D materials are completely distinct from their bulk counterpart. The band gap, electron mobility, and contact resistance change very rapidly as the bulk semiconductors thinned down to few-layer to monolayer thickness [2, 3]. Since the isolation of graphene (the mother of all 2D material) in 2004 by Prof. Geim and Novoselov, numerous other 2D materials have been explored for their wide range of applications in nano-electronic and photonic smart devices.

Similar to graphene, several other 2D materials have extensively been studied and grabbed huge attention with their unique electronic, optical, mechanical, and chemical properties [4–6]. These 2D materials outperform conventional materials primarily due to the availability of easily tunable properties in the former, which is an essential ingredient for modern smart devices. The intrinsic characteristics of these materials could easily be tailored by strain engineering, phase engineering, defect engineering, forming heterostructures, changing the number of layers, or modifying the surface morphology. The 2D materials possess negligibly small dimensions in one direction. For example, mechanically exfoliated single layers of graphene and MoS_2_ have a thickness of 3.4 and 6.5 Å, respectively. The practical applications of traditional devices are limited by the inherent properties of conventional materials. For instance, conventional devices are rigid and lose their mechanical and electronic attributes upon bending. The natural flexible and excellent transparent nature of atomically thin materials overcome these limitations and make them promising candidates for foldable and wearable flexible devices [7, 8]. In recent years, stretchable smart devices have also been developed using 2D materials as these materials can withstand high deformation without losing their inherent characteristics. Smart wearable fabrics with tuneable properties could be designed for environmental monitoring, complex computing, and flexible Internet of Things (IoT) applications.

Nowadays, 2D metals, layered magnetic materials, van der Waals (vdW) heterostructures, 2D semiconductors, and insulators are the most commonly used building blocks for developing smart electronic devices. These smart devices have been thoroughly explored for photodetectors, light-emitting diodes, gas sensors, energy storage devices, and [9–12]. For example, graphene could be used as the channel region as well as for making electrical contacts. Hence, by using only graphene, smart devices could be fabricated having superb mechanical flexibility and transparency without compromising the electronic behavior of devices. These devices could easily switch between two or more different states to meet the desired requirement.

**Fig. 1:**
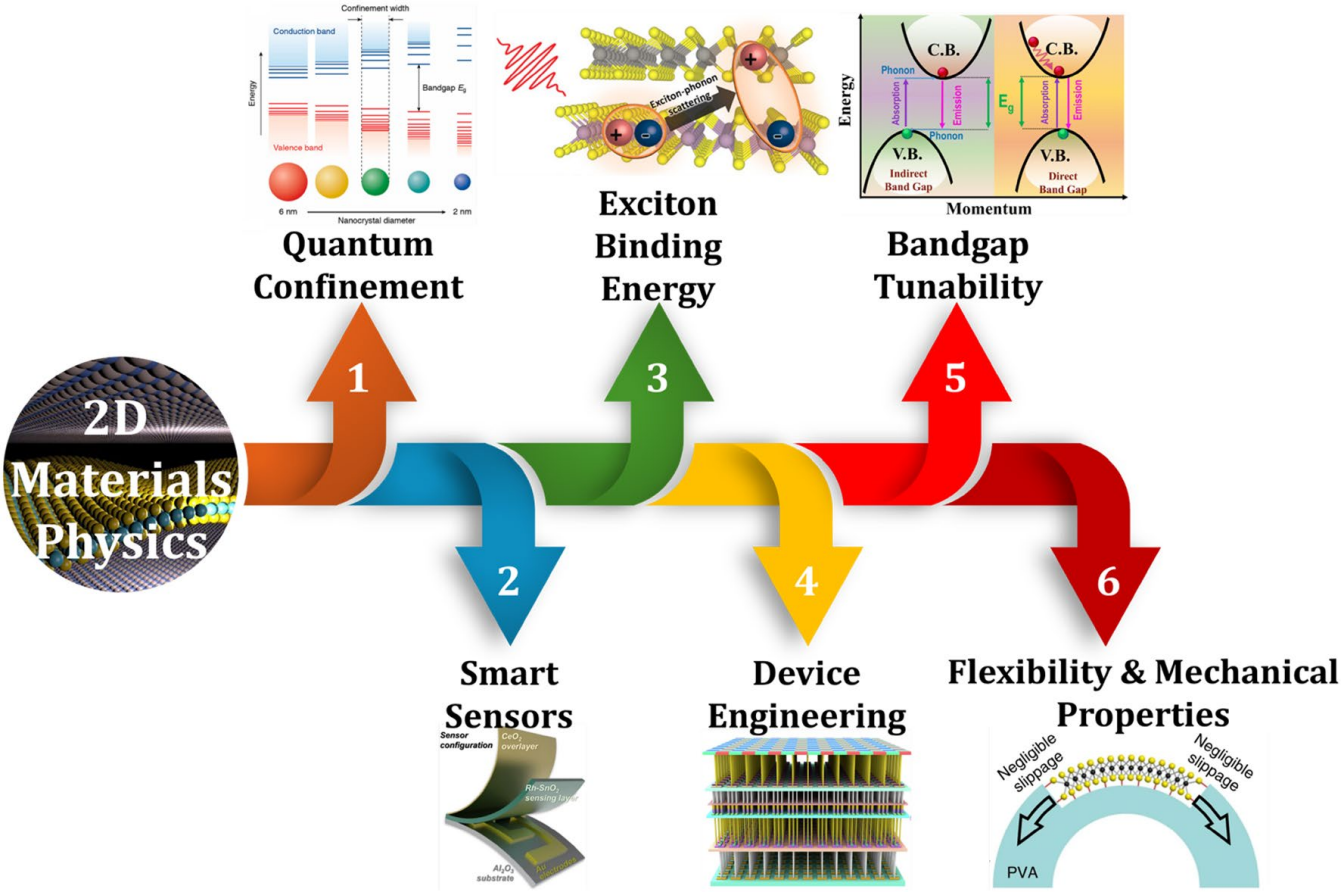
Engineering 2D materials for developing smart devices. Reproduced with permission [13–17]. Copyright (2011), American Chemical Society

In this review, we discuss the unique and exciting physics of 2D materials facilitating the transfer of traditional devices to smart devices, as shown in Fig. 1. We have also discussed some of the inherent bottlenecks existing in conventional materials limiting their uses for modern technological applications. We started by examining the role of 2D materials in modern electronic devices. Then, we highlighted the recently used strategies for developing smart devices. We shed insight into the physics of various techniques in improving the performance of pristine 2D materials-based devices. Further, we examine the role of device architectures serving as the foundation of advanced technological applications. The subsequent section focuses on potential applications of 2D materials including wearable, biomedical, quantum, and energy storage applications. Finally, we wrap up this review by giving an outlook on future opportunities and the associated technological implications for developing smart devices using 2D materials (Table 1).

**Table 1:** Unique physical properties of 2D materials used for various applications

Material	Physical properties	Applications
Graphene	Fast carrier mobility	Ultrafast photodetectors [24] and lasers [25]
	Bandgap:0 eV	Broadband photodetectors[26] (UV to infrared)
	Ultrafast carrier relaxation time	High-speed optical communication [27], imaging in security [28], and medical diagnostics [29]
	Exceptionally low noise	Detection of single gas molecules [30]
	High tensile strength (~ 130 GPa)	Aerospace and automotive industry [31]
	High surface area (~ 2630 m^2^ g^−1^)	Energy storage devices like supercapacitors and batteries [32]
TMDs	Tunable bandgaps (Bandgap: 1–2.5 eV)	Low-power, high-efficiency digital electronics [33]
	High absorption coefficient (absorption up to 20% of incident light)	Solar cells [34] and photodetectors [35]
	Strong spin–orbit coupling	Spintronic [36] and valleytronic [37]
	High surface reactivity	Hydrogen evolution reaction (HER) in water splitting [38]
Hexagonal Boron Nitride (h-BN)	Atomically flat surface	Surve as perfect substrate for epitaxial growth of other 2D materials [39]
	Large bandgap (~ 5.9 eV)	Excellent electrical insulator [40]
	High mechanical strength	Protective coating and support material for other 2D materials [41]
	High breakdown voltage	Gate dielectrics in transistors [42]
	Resistance to radiation damage	Ideal for space applications and nuclear technology [43]
Phosphorene	Tunable bandgaps (Bandgap: 0.3–2 eV)	Solar cells [44] and spintronics [45]
	Ambipolar conduction	Used in optoelectronics and logic circuits for efficient charge transport [46]
	Strong light absorption	Useful in photodetectors and solar cells [47]
	Photoluminescence	Light-emitting diodes (LEDs) and lasers [5]
	Anisotropic elastic properties	Direction-sensitive strain sensors [48]

## Device Scaling Using 2D Materials

Smart devices differ from traditional electronic devices primarily in functionalities, miniaturization, automation, and data processing capabilities. Bulk silicon (Si) is the primary constituent of traditional electronic devices. These conventional devices offer reliable and efficient operation in memory devices, MEMS sensors, telecommunications, and mixed-signal processing. However, they failed as the device sizes scaled down to the atomic scale. These devices also suffer from limited flexibility due to the material's intrinsic properties, making them less adaptable for developing flexible smart devices. Data processing is one of the prime components in evaluating the performance of smart devices. The newly emerging fields such as artificial intelligence and the Internet of Things demand very high computational power and efficient energy-based electronic devices to perform multiple tasks simultaneously. The silicon transistor technology tried to meet these demands by continuously scaling down its size and embedding memory components into the device architecture. Some of the architectural innovation has also been introduced into the conventional technology for developing smart devices. FinFETs, multigate, and nanowire structures allow to reduce the gate length to 10–15 nm without the occurrence of short channel effects. However, material innovation is required to further reduce the channel length below 5 nm [18].

The unique properties of 2D materials such as atomic thickness, high surface-to-volume ratio, bandgap tunability, and mechanical flexibility bring transformative functionalities beyond the capabilities of conventional Si devices. The increasing computation demands the miniaturization of transistors beyond the limits of Moore's law which were impossible to achieve with conventional materials due to scaling limitations and short-channel effects [1]. A large number of smart devices require stretching, bending, and folding to adapt various surfaces for flexible and wearable electronics applications. However, traditional materials are not suitable for these applications paving the way for using 2D materials. Additionally, the 2D materials possess atomic thickness resulting in a very high surface-to-volume ratio, enabling them highly suitable for ultrasensitive chemical sensors used in environmental and health monitoring applications.

The scaling boosts the performance of devices by enhancing computational power, reducing power consumption, and lowering cost per operation. Field effect transistors are one of the most important foundational blocks of digital electronic devices. In conventional silicon technology, as we reduce the gate length below 5 nm, the leakage current becomes very high, and other short channel effects also start dominating [19]. Hence, limiting the scaling of traditional silicon-based transistors. With the introduction of 2D materials, these transistors could be shrunk down to molecular scale without compromising the device's performance. Over the last decade, MoS_2_, one of the well-known 2D materials, has emerged as a potential candidate for substituting silicon technology due to its exceptional inherent attributes. Desai et al. have shown MoS_2_ transistors possessing a gate length as low as 1 nm [20]. The device demonstrated an excellent on/off ratio of 1 × 10^6^, a perfect subthreshold swing of 65 mV dec^−1^, and a very low value of leakage current.

The carrier mobility of conventional 3D bulk materials decreases drastically upon thinning down due to pronounced interfacial scattering effects. When scaling starts reducing the dimension of the device beyond a particular limit (< 7 nm), the quantum effects become more dominating, and hence devices behave unexpectedly and fail to meet the desired objectives. On the other hand, 2D materials intrinsically possess atomic thin flat surfaces with a very high value of carrier mobility. Elias et al. have demonstrated mobility as high as 10^6^ cm^2^ V^−1^ s^−1^ in graphene at a temperature of 2 K [21]. Similarly, Long et al. have shown ultrahigh carrier mobility in a field-effect transistor comprised of a few nanometer-thick layers of black phosphorus [22]. They reported a hole mobility of 45,000 and 5200 cm^2^ V^−1^ s^−1^ at cryogenic temperature and room temperature, respectively. Wu et al. have synthesized atomically thin 2D bismuth oxyselenide (Bi_2_O_2_Se) by a highly controllable chemical vapor deposition technique (CVD) [23]. The CVD-grown Bi_2_O_2_Se showed ultrahigh mobility of more than 20,000 cm^2^ V^−1^ s^−1^ at 2 K and a fairly large value of 313 cm^2^ V^−1^ s^−1^ at room temperature. Hence, the superb motilities of 2D materials even at atomic dimensions facilitate scaling of devices up to a large extent without compromising the performance. Moreover, multifunctionality is one of the prime requirements for smart devices. 2D materials exhibit phase transitions from one state to another when exposed to external stimuli allowing different applications under different conditions. The quantum confinement effects in 2D materials originate tunable bandgaps and nonlinear optical effects making them useful for quantum LEDs, single-photon emitters, photodetectors, and solar cells. For a better understanding, we presented key physical properties and performance data of typical 2D materials in a tabular form, in the revised manuscript.

The statistical physics of 2D materials delves into their unique thermodynamic behaviors and transport phenomena, which are fundamentally influenced by their reduced dimensionality. In terms of heat capacity, the phonon density of states in 2D materials differs significantly from bulk systems, as in-plane vibrational modes dominate. For instance, graphene exhibits an unusual linear temperature dependence of specific heat at low temperatures, deviating from the cubic law observed in 3D systems. Additionally, 2D materials like graphene have exceptionally high thermal conductivity, surpassing 3000 W m^−1^ K^−1^ [49], due to long phonon mean free paths and minimal scattering. This property makes them ideal candidates for thermal management applications. Transport phenomena in 2D systems can be described using Boltzmann transport theory, which accounts for charge and heat transport processes [50]. 2D materials exhibit high carrier mobility under low scattering conditions; however, defects, impurities, and edge roughness can significantly impact their electrical properties. For example, pristine graphene's carrier mobility can exceed 200,000 cm^2^ V^−1^ s^−1^ [51], but decreases in the presence of defects, which can be studied statistically to model degradation effects. Moreover, phase transitions in 2D materials also exhibit unconventional behaviors due to unique dimensional constraints. The Mermin–Wagner theorem predicts the suppression of long-range order in 2D systems due to thermal fluctuations, particularly in isotropic systems [52]. However, 2D materials having magnetic characteristics like CrI₃ defy this prediction due to spin–orbit coupling and anisotropy, enabling the realization of 2D ferromagnetism.

On the quantum scale, 2D materials exhibit remarkable phenomena due to confinement effects. Their band structure is profoundly altered compared to their 3D counterparts. For instance, graphene features a linear dispersion near the Dirac points, resulting in massless Dirac fermions [53]. This property allows electrons to travel, leading to extremely high conductivity and the absence of backscattering. Similarly, transition-metal dichalcogenides (TMDs) like MoS₂ transition from an indirect to a direct bandgap in their monolayer form, which is crucial for optoelectronic applications such as light-emitting diodes (LEDs) and photodetectors. Quantum Hall effects (QHE) in 2D materials result from their electronic band structure and topology. In graphene, the integer QHE manifests at room temperature due to its robust topological properties, while fractional QHE has been observed in other 2D electron gas systems under strong magnetic fields. Additionally, the Berry phase in graphene (π phase) plays a crucial role in its electronic transport, resulting in weak localization and interference phenomena [54]. The interplay of excitons and many-body interactions is another quantum hallmark of 2D materials. Reduced dielectric screening in TMDs leads to high exciton binding energies, significantly higher than in bulk materials. This strong binding enhances light-matter interactions, essential for photonic and optoelectronic devices. Excitonic effects also lead to fascinating quantum behaviors such as Mott transitions, where excitons dissociate into free charges under high carrier densities.

Quantum transport phenomena in 2D materials reveal unique behaviors due to dimensional constraints. Klein tunneling in graphene demonstrates perfect transmission of electrons through potential barriers, due to its relativistic-like Dirac fermions [55]. In TMDs, spin-valley coupling links the spin and valley degrees of freedom, enabling the emerging field of valleytronics, where valley states are manipulated for information processing. These statistical and quantum properties make 2D materials highly promising for a wide range of applications, from electronics and photonics to quantum computing and energy storage. By exploring and leveraging these unique characteristics, researchers can develop new technologies that surpass the limitations of traditional materials.

## Strategies for Developing Smart Devices Using 2D Materials

The evolution of functionalities in electronic devices has led to the development of smart devices. However, the use of conventional devices could not address the ever-increasing performance demand. New strategies are required to explore the new functions and advanced principles of recently discovered 2D materials. The discovery of these strategies upgraded traditional devices into smart devices. These novel devices offer better control over the functionality of the device while reducing the power and size requirements. In recent years, these strategies have attracted huge interest from researchers to solve the existing technological challenges. For instance, changing the phase of the material converts it into metallic from its semiconducting behavior, suitable doping of an element changes the conductivity of the materials drastically, choice of suitable substrate offers epitaxial growth, and introducing defects in grown materials assists in tuning their properties as per the requirements of specific applications. Here, in this section, we will discuss some of the most widely used strategies for developing smart devices.

### Phase Transition

The exploration of new phases of materials has been of great interest in making them suitable for specific applications. Controlling the phase of 2D materials through external stimuli has proved to be an effective tool for developing smart devices. The phase transition of single 2D materials modulates their properties to meet specific needs. There are numerous ways such as chemically driven transition, thermally induced transition, strain-induced transition, laser-induced transition, electric-field induced transition, and electrostatic-doping induced transition for developing different phases of 2D materials [56–60]. For instance, MoS_2_ undergoes a phase transition from 2H to 1T phase upon alkali metal intercalation by changing its coordination from trigonal prismatic to octahedral [61]. This structural change occurs due to charge transfer from the alkali metal to the transition-metal d orbitals. Hence, depending upon the requirement, MoS_2_ could work as a semiconductor in the 2H phase or could be used as a metal in the 1T phase.

**Fig. 2:**
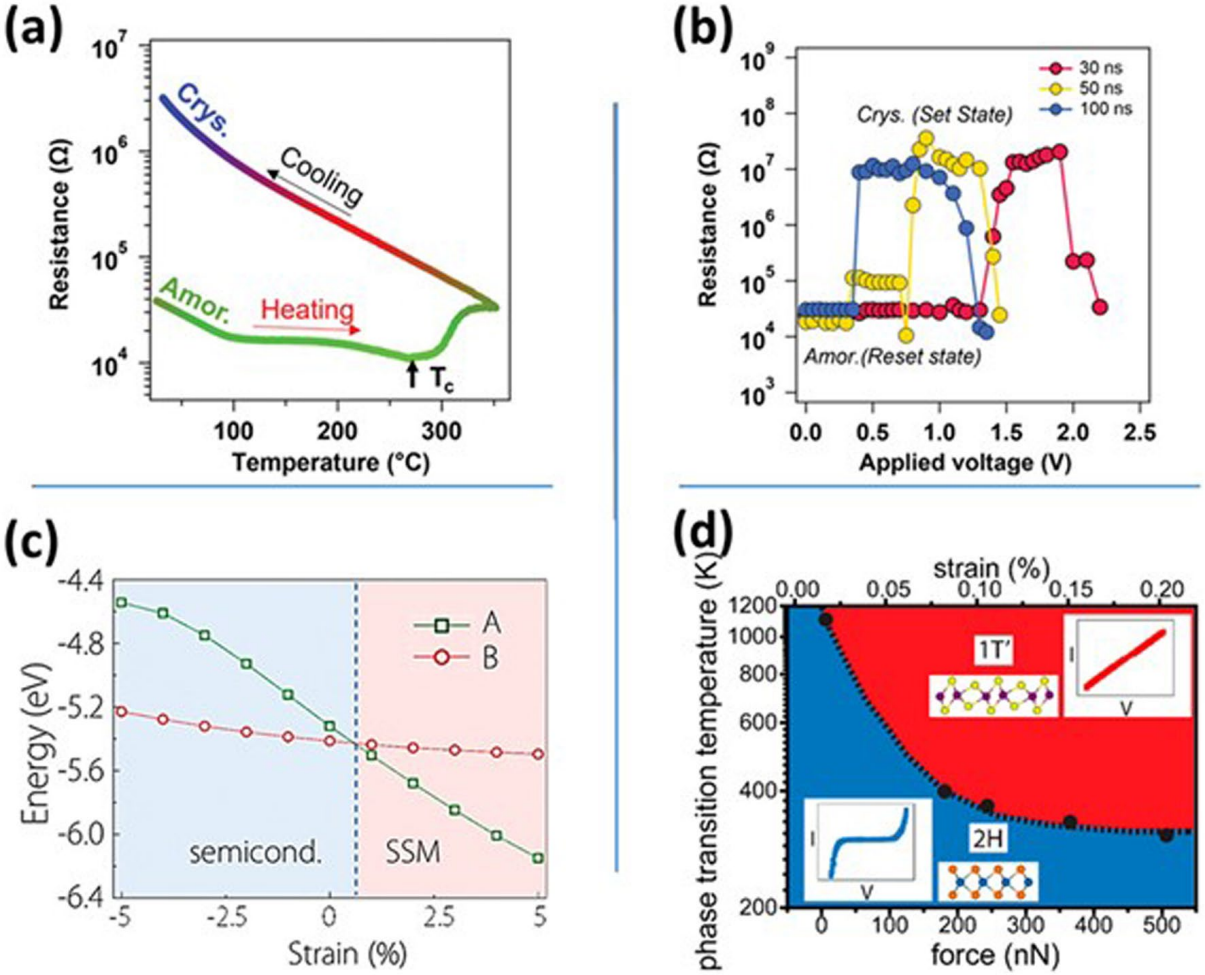
**a** Resistance variation of amorphous and crystalline phases of NbTe_4_ with varying temperatures. **b** Resistance variation with applied voltage indicating switching between set and reset state of NbTe_4_ based memory cell. Panels **a** and **b** are reproduced with permission [66]. Copyright (2023), Wiley–VCH. **c** The transition of 2D blue phosphorene oxide from the semiconducting phase to the symmetry-protected semimetal (SSM) phase upon applying moderate strain. Reproduced with permission [67]. Copyright (2016), American Chemical Society. **d** MoTe_2_ phase transition between semiconducting (2H) and metallic (1T′) as a function of temperature and force. Reproduced with permission [68]. Copyright (2015), American Chemical Society

Temperature also works as a potential external stimulus to drive the phase transition. Similar to MoS_2_, another 2D material MoTe_2_ could make the phase transition from 2H to 1T′ at high temperature due to higher entropy of 1T′ phase [62, 63]. In addition to temperature, laser heating could also be used for MoTe_2_ phase transition due to the generation of Te vacancies [64]. Yang et al. have also demonstrated a temperature-dependent phase change memory using MoTe_2_ films [65]. The resistive switching originated from the phase transition of MoTe_2_ from semiconducting (2H) to metallic (1T′). Shuang et al. have reported phase-change random access memory using NbTe_4_ [66]. Amorphous NbTe_4_ was grown through the sputtering technique and converted to crystalline form by post-annealing. The device offers better thermal stability and reduced energy requirements due to the low melting point and high crystallization temperature of NbTe_4_. The sheet resistance of NbTe_4_ was significantly higher in crystalline form compared to amorphous form, as shown in Fig. 2a. The resistance versus voltage characteristics of NbTe_4_-based memory cells with varying pulse widths are displayed in Fig. 2b. At smaller voltages, the phase of NbTe_4_ remains the same due to the low value of Joule heating energy. However, the phase changes to crystalline with a further increase in voltage resulting in a wider set/reset window.

In recent years, strain engineering has also emerged as one of the most potential techniques for reversible and controlled phase transitions. This technique is widely used in flexible devices to make them suitable for various applications by controlling their physical and electronic properties. Zhu et al. have reported a semiconductor-to-semimetal phase transition in blue phosphorene oxide upon applying a moderate strain, as depicted in Fig. 2c [67]. A very small tensile strain of 0.6% reduces the direct bandgap to zero while compressive strain increases the bandgap of the material. In another work, Song et al. have demonstrated that upon applying a very low tensile strain (0.2%), the MoTe_2_ makes a transition from semiconducting (2H) to metallic (1T′) phase and hence enhances the conductance by 10,000 times (Fig. 2d) [68].

The phase change in 2D materials in the presence of external stimuli could be explained by Landau phase transition theory [69]. In response to a large external electric field, magnetic field, temperature, pressure, or strain, the 2D materials form quantized Landau levels. In 2D materials, properties of these quantized energy levels have been studied using scanning tunnelling spectroscopy and electron transport measurements. Landau's model explains the phase change in 2D materials through order transitions reflecting symmetry-breaking phenomena. First-order and second-order phase transition occurs in 2D materials resulting in different physical properties of the systems, such as such as the metal–insulator transition in TMDs. The unconventional valley-dependent Landau levels transition in graphene, silicene, and TMDs possess valley-dependent orbital moment. Ju et al. have reported valley-dependent optical transitions in bilayer graphene by examining inter-Landau levels transitions [70]. In another work, Chen et al. demonstrated a bandgap of ~ 38 meV in epitaxial graphene/h-BN heterostructures by analyzing the Landau level transitions [71]. Moreover, the Landau level transitions play a crucial role in exploring the fundamental physics of 2D materials. These Landau-level transitions could be controlled by magnetization, electron density, or lattice distortion to unlock novel functionalities in next-generation smart devices.

In summary, phase transition strongly affects the physical, chemical, and electronic properties of 2D materials, offering a wide tunability for various applications. For instance, in TMDs the phase transition between 2H and 1T modifies the material properties from semiconducting to metallic state, enabling them for device engineering. Moreover, phase transitions modulate the bandgap of 2D materials changing the light absorption and emission properties in photodetectors. Highly sensitive broadband photodetectors with improved figures-of-merit could be designed using phase transition. In addition, combining phase transitions with other strategies, such as doping and functionalization offers an extra edge for tailoring the intrinsic properties of 2D materials due to the synergistic effects.

### Elemental Doping

Non-destructive doping is one of the primary challenges in tuning the properties of 2D materials for developing smart devices. A particular amount of suitable dopant changes the carrier concentration and controls the electrical current in electronic and optoelectronic devices. Efficient doping results in desirable properties of materials for specific applications without any structural damage. Several types of doping techniques for 2D materials have been introduced for developing smart devices, for instance, ion implantation, electrostatic doping, chemical doping, molecular doping, and substitutional doping are some of the most widely used doping techniques. However, ion implantation poses some serious challenges in 2D materials as the bombarding of doping elements could damage the structure of materials inducing undesirable properties. The uses of electrostatic and molecular doping for smart devices are not suitable due to electrical breakdown, complexity of fabrication, and molecular instability. Therefore, chemical and substitutional doping emerge as the most effective techniques for elemental doping. Yoo et al. have examined the effect of chemical doping in multilayer MoS_2_ [72]. The doping of poly(diketopyrrolopyrrole-terthiophene) (PDPP3T) improved the MoS_2_ transistor on current by a factor of 4.6 with a current on/off ratio of 10^6^.

Doping involves donating or accepting electrons to manipulate the charge density of the host semiconductors. A dopant could work as a donor or acceptor depending on its highest occupied molecular orbital (HOMO) and lowest unoccupied molecular orbital (LUMO) levels relative to the Fermi level of the material. Apart from the required doping, the substrate trap states could also modify the Fermi level of the material due to localized energy states. Broadly, doping in 2D materials could be categorized into two: substitutional doping and surface charge transfer doping [73, 74]. In substitutional doping, one or more atoms of the host material get substituted by the dopants. The substitutional doping could disturb the original structure of the 2D material. On the other hand, when doping is obtained by electron transfer between 2D materials and dopants, it is called surface charge transfer doping. One of the primary advantages of surface charge transfer doping is that it cannot damage the structure of the material. Moreover, surface charge transfer doping offers a reversible process through the desorption of dopants from the material surface. However, substitutional doping is an irreversible process due to the presence of strong chemical bonds between host material and dopants.

**Fig. 3:**
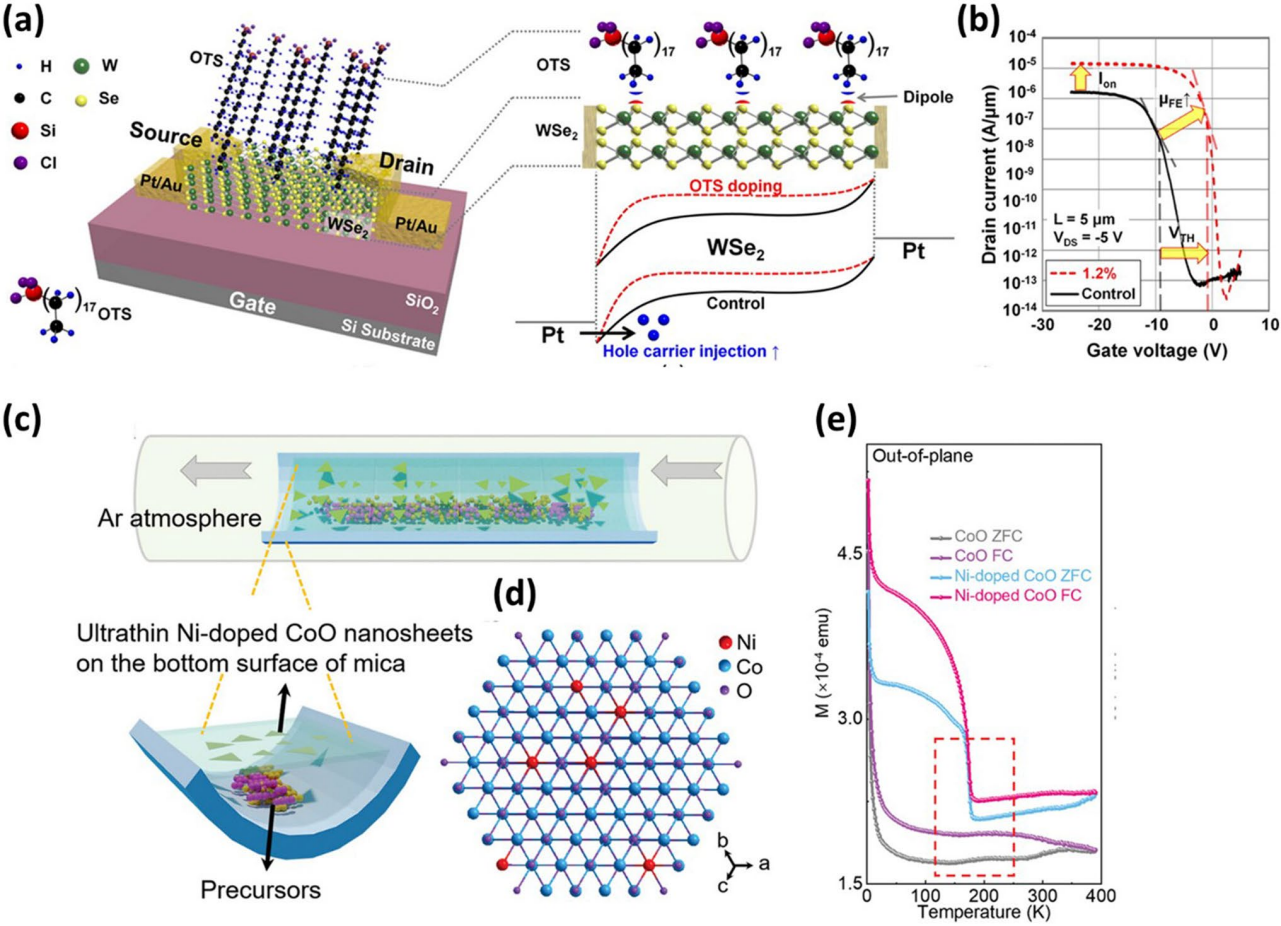
**a** Schematic of octadecyltrichlorosilane (OTS) doped WSe_2_ transistor and energy band diagram of pristine and doped WSe_2_ at the metal–semiconductor interface. **b** Current–voltage characteristics of pristine and OTS-doped WSe_2_ transistor. Panels **a** and **b** are reproduced with permission [75]. Copyright (2015), American Chemical Society. **c** Schematic illustrating growth of CoO nanosheets via atmospheric pressure chemical vapor deposition technique. **d** Hexagonal atomic representation of CoO nanosheet depicting Ni-doping along the [107] axis. **e** Magnetic susceptibility of CoO and Ni-doped CoO at different temperatures under a magnetic field of 0.1T. Panels **c**-**e** are reproduced with permission [76]. Copyright (2023), Wiley–VCH

Most of the earlier research has reported a high level of doping in TMDs, converting their semiconductor behavior near to metallic. Therefore, controlled doping in 2D materials is essential to tune their properties as per the application requirement. Kang et al. have reported a controllable non-degenerate p-type doping process using octadecyltrichlorosilane (OTS) in WSe_2_-based transistor [75]. The OTS consists of a methyl (-CH_3_) group, which decreases the electron concentration in the channel. Figure 3a displays the schematic of a p-doped WSe_2_ transistor with an energy band diagram of undoped and doped transistors under negative drain biasing. The band diagram of doped WSe_2_ moves upward resulting in increasing the electric field and lowering the Schottky barrier. Therefore, the hole carrier injection at the Pt-WSe_2_ interface increases through tunnelling, as depicted in Fig. 3a. The p-type doping could easily be controlled between 2.1 × 10^11^ and 5.2 × 10^11^ cm^−2^ facilitating optimization of threshold voltage, carrier mobility, and on/off current of the transistor. To assure p-type doping, *I*_D_–*V*_G_ characteristics of undoped and doped transistors were examined with varying OTS concentrations from 0.024% to 1.2%, as shown in Fig. 3b. The threshold voltage increases by 8.65 V upon 1.2% p-type doping as compared to the undoped transistor. Moreover, the on-current is also enhanced by a factor of 10, increasing the mobility of the transistor.

Elemental doping also proved to be an effective technique for tuning the magnetic properties of 2D materials. Jiang et al. have demonstrated the occurrence of a ferromagnetic nature in cobalt monoxide (CoO) upon nickel doping, while pristine CoO showed nonferromagnetic behavior [76]. The Ni-doped CoO was grown in the mica substrate by a van der Waals epitaxy technique, as shown in Fig. 3c. In the Ni-doped structure, Co atoms were replaced by Ni atoms without disturbing the original structure as both the atoms possess similar atomic radiuses (Fig. 3d). The absence of dangling bonds on the mica substrate facilitates the lateral growth of 2D material. To examine the magnetic behavior of Ni-doped CoO, zero field cooling (ZFC) and field cooling (FC) magnetization have been studied with varying temperatures, as displayed in Fig. 3e. The ZFC and FC of doped samples showed ferromagnetic behavior with a transition at 174 K. Therefore, the magnetism in 2D materials could be effectively controlled by tuning the elemental doping.

Therefore, the incorporation of suitable dopants in 2D materials modulates the electrical conductivity, optical absorption, and mechanical strength of 2D materials. Elemental doping either increases the electrical conductivity or hole concentration depending on the doping type, making 2D materials suitable for designing transistors, diodes, and other nano-electronic devices. Doping also creates mid-gap states or changes the excitonic behavior, hence influencing the photovoltaic performance of optical devices. Continuous refinement of doping techniques promises to achieve higher efficiency, scalability, and versatility, driving innovations in next-generation material technologies.

### Substrate Engineering

Substrate engineering is one of the most commonly used techniques for developing smart devices. The choice of the substrate significantly influences the 2D material nucleation and its epitaxial growth. The uniformity of the synthesized 2D materials is also largely dependent on the substrate. The inherent properties of substrate such as thermal expansion coefficient, surface roughness, and catalytic properties severely affect the quality of grown 2D materials. The mobility, orientation, bandgap, and other electronic properties of the deposited materials could be controlled by choosing a suitable substrate. For instance, 2D hBN works as a perfect substrate facilitating single crystalline growth of other 2D materials resulting in high-performance devices possessing exceedingly high carrier mobilities due to its ultra-flat nature [77, 78].

**Fig. 4:**
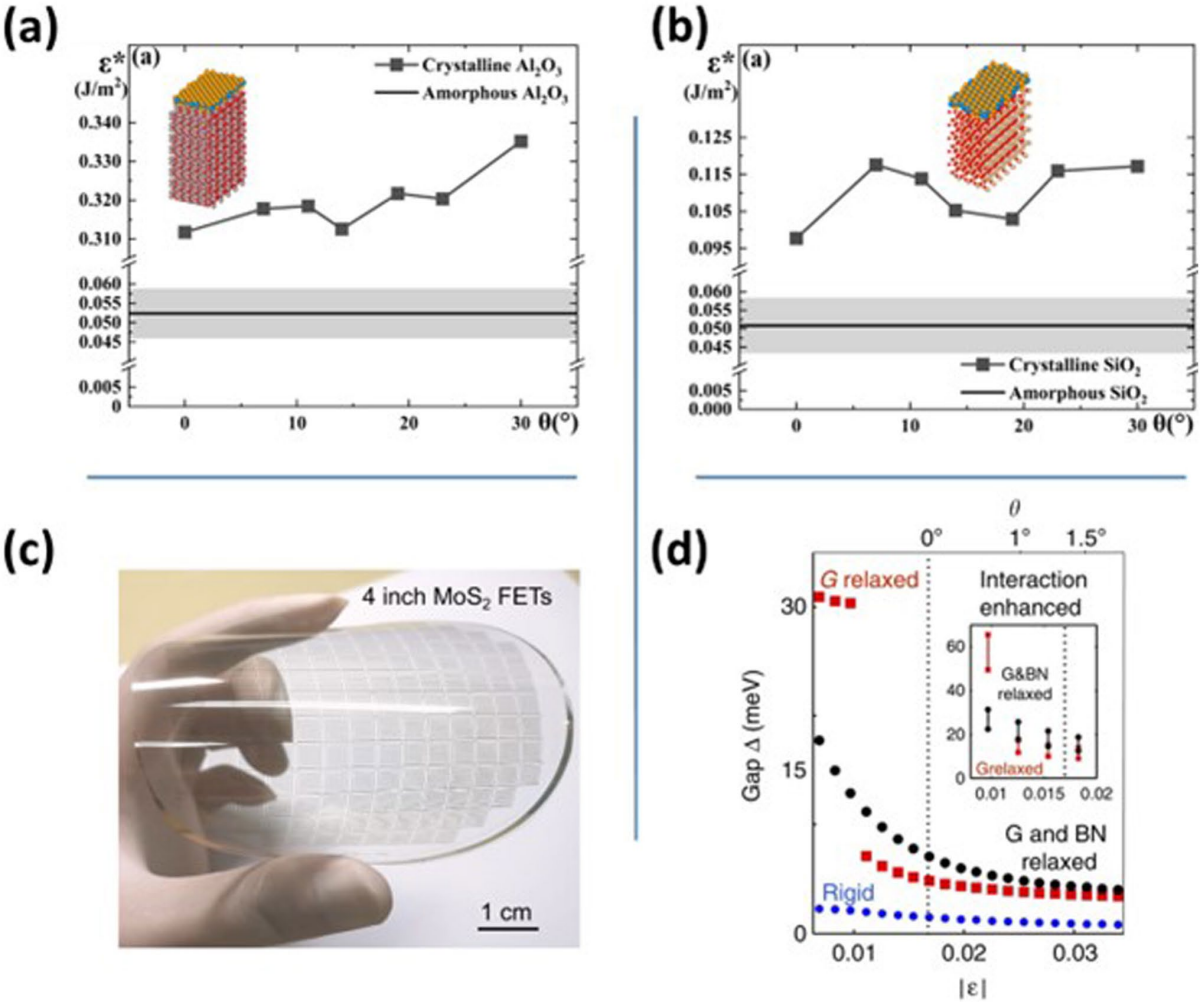
Binding energy of 2D WSe_2_ film at different rotation angles grown over crystalline and amorphous. **a** Al_2_O_3_ and **b** SiO_2_ substrates. Panels **a** and **b** are reproduced with permission [79]. Copyright (2024), American Chemical Society. **c** Flexible wafer-scale MoS_2_ thin-film transistors on polyethylene terephthalate (PET) substrate. Reproduced with permission [81]. Copyright (2023), Wiley–VCH. **d** Energy gap versus lattice constant mismatch when graphene and boron nitride both are rigid, when both are relaxed and when only graphene is allowed to relax. In the inset, electron–electron interactions are also taken into account. Reproduced with permission [82]. Copyright (2015), Springer Nature

The careful selection of substrate plays an instrumental role in the post-growth processes for developing smart electronic devices. Momeni et al. have studied the impact of various substrates on WSe_2_ monolayer orientation, by theoretical and experimental techniques [79]. The study was conducted on crystalline and amorphous Al_2_O_3_ and SiO_2_ substrates. Figure 4a, b shows the binding energy (ε*) of single-layer WSe_2_ grown on various substrates. The minimum binding energy was achieved when the WSe_2_ orientation angle was 0° on crystalline Al_2_O_3_ and SiO_2_ substrates. However, the binding energy remained nearly constant at 0.052 ± 0.0065 and 0.051 ± 0.008 J m^−2^ in the case of amorphous Al_2_O_3_ and SiO_2_ substrates, respectively. The binding energies on amorphous substrates are lower as compared to their crystalline counterpart due to a lack of well-defined structures. The low binding energy on a non-crystalline substrate facilitates polycrystalline growth of 2D materials. Moreover, the grown 2D film could easily be separated from the amorphous substrates to form complex structures owing to the lower value of binding energies.

The carrier mobility in 2D materials is significantly influenced by the presence of grain boundaries. To address this issue, researchers have synthesized single-crystal material on c-plane sapphire substrates. Wang et al. have epitaxially grown single-layer MoS_2_ over the entire 4-inch sapphire wafer using an optimized chemical vapor deposition technique [80]. The monolayer MoS_2_ exhibits excellent electronic properties with carrier mobility of ∼70 cm^2^ V^−1^ s^−1^ and a current on/off ratio of ∼10^9^. Recently, to develop energy-efficient wearable electronic devices, Tang et al. have grown wafer-scale single-layer MoS_2_ over the 4-inch flexible polyethylene terephthalate (PET) substrate (Fig. 4c) [81]. The flexible device performance was comparable to rigid MoS_2_-based transistors with average carrier mobility and a current on–off ratio of ~ 70 cm^2^ V^−1^ s^−1^ and 5 × 10^7^, respectively.

The bandgap of 2D materials could also be tuned effortlessly by growing them on different substrates due to strain engineering. Except graphene, most of the 2D semiconducting materials possess bandgaps making them suitable for terahertz to ultraviolet range of applications. Jung et al. have demonstrated a bandgap of ~ 20 meV in graphene by placing it on a hexagonal boron nitride (hBN) substrate, as shown in Fig. 4d [82]. The carbon atoms in graphene get relaxed when placed at the hBN surface, resulting in the introduction of a bandgap.

In addition to substrate engineering, surface energy engineering also plays a crucial role in controlling the surface and interfacial properties of 2D materials. The surface traps, oxygen vacancies, and nanostructures' domain orientation at the surface or the interface could easily be modified for developing surface-responsive electronic devices. Due to vast variety of applications, several strategies have been adopted for surface energy modification, such as the ion beam process, self-assembly technique, and cross-linking of copolymer films [83]. A surface energy modifier could also be used to improve the stability and efficiency of perovskite solar cells. Su et al. have used heptadecafluorooctanesulfonate tetraethylammonium (HFSTA) to modify the surface of the TiO_2_ electron transport layer [84]. The HFSTA improved the carrier-extraction efficiency by lowering the surface energy. The modified TiO_2_ results in superior crystalline perovskite film with an efficiency of more than 25% due to less number of heterogeneous nucleation sites.

In summary, substrate engineering provides a versatile platform for tuning the properties of 2D materials. The selection of substrate changes the electronic, optical, and mechanical properties of 2D materials by controlling the interfacial interactions, strain, and surface roughness. The substrate-induced strain changes the bandgap of the material while the charge transfer between the substrate and grown 2D materials enables the doping effects. High dielectric substrates can screen Coulomb interactions in 2D materials facilitating high carrier mobility and reducing charge trapping at the surface and interface. While integration of 2D materials with flexible substrates finds applications in flexible and wearable electronics. Moreover, plasmonic substrates improve the light-matter interactions, enhancing Raman scattering and photoluminescence of optical devices.

### Defect Engineering

Structural defects are imperfections that break the periodicity of regularly arranged atomically thin 2D materials. These defects have been intentionally engineered in 2D materials to make them suitable for various applications including biomedical, quantum technologies, and energy storage devices. Effective defects could easily be created in 2D materials as most of their atoms are exposed for engineering. For instance, introducing defects in graphene opened a bandgap, making it suitable for switching applications. These defects severely impacted the electronic, optical, chemical, and mechanical properties of the materials. Some of these defects affect the device's performance favorably, while others may affect it adversely. Therefore, exploring the defect nature and their distribution across 2D materials is essential for the transition toward smart devices.

**Fig. 5:**
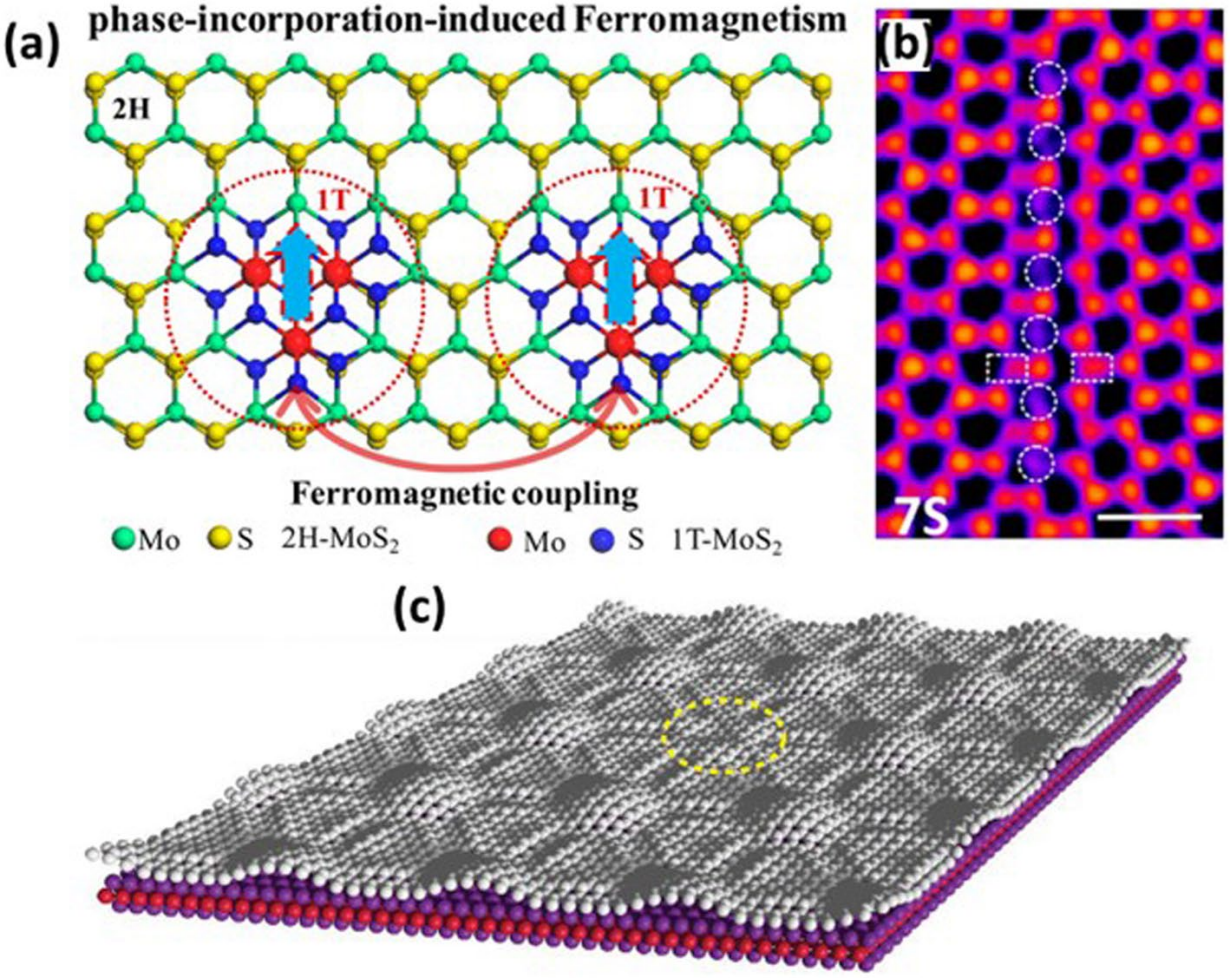
**a** Schematic illustration of occurrence of ferromagnetism in nonmagnetic MoS_2_ nanosheets via phase incorporation. Reproduced with permission [85]. Copyright (2015), American Chemical Society. **b** TEM image displaying one line of single S vacancies consisting of 7 S vacancies. Reproduced with permission [86]. Copyright (2016), American Chemical Society. **c** Three-dimensional view of vacant hill model representation of deposited graphene on Ru substrate with a stacking fault. Reproduced with permission [87]. Copyright (2020), IOP Publishing Ltd

Structural defects in 2D materials could be divided into three categories: (1) Point or 0D defects; (2) line of 1D defects; and (3) 2D or planer defects. Point defects include intrinsic and extrinsic defects. The intrinsic defects are generated due to the absence of atoms at their original site resulting in vacancies. While extrinsic defects occur due to heteroatom doping or when an atom occupies an adjacent interstitial site. Cai et al. [85] have induced sulfur vacancies in MoS_2_ through a two-step hydrothermal process. The sulfur vacancies facilitate 1T phase incorporation into the 2H phase of MoS_2_ as illustrated by Fig. 5a. This transformation introduces ferromagnetism in nonmagnetic MoS_2_ at room temperature.

Line defects stem from different lattice orientations of various grains. Moreover, 2D materials grown by various synthesizing techniques possess grain boundaries leading to variation in band structure and electrical conductivity due to changes in chemical stoichiometry. However, line defects are not fixed in 2D materials, impacting the mobility of host materials. Wang et al. have shown the appearance of metallic tracks in monolayer MoS_2_ due to line defects [86]. The defect structures cause lattice compression and it increases with an increase in the length of line defect. Hence, the atomic distance between S and Mo atoms changes. Figure 5b shows the TEM image of line defects containing seven S vacancies. The DFT calculations showed that the distance between two Mo atoms compresses to 4.2 Å in the case of seven S vacancies from its original distance of 5.3 Å in the case of pristine configuration. Similarly, the atomic distance between Mo and S atoms changes in case of line defects offering precise control of the semiconductor/ metal interface.

Planer defects occur when a 3D structure is divided into smaller domains of symmetric atomic arrangements. These defects exist at the boundary between neighboring domains. The planer defects in 2D materials primarily consist of stacking faults. Artaud et al. have studied the nature of vacant hills in graphene [87]. This defect periodically appears on epitaxially grown graphene on a metallic substrate with a moiré pattern, as shown in Fig. 5c. High-quality pristine graphene requires the removal of these vacant hill defects to improve its inherent characteristics such as mobility. However, engineering these defects might discover novel optical and spin properties in graphene.

Defect engineering could be done through several techniques including ion irradiation, chemical and thermal treatment, and strain engineering. However, in recent years, ion irradiation has been proven to be one of the most useful defect engineering techniques for controlling the properties of atomically thin 2D materials [88]. The ion irradiation changes the surface morphology and reduces the vdW interlayer spacing and hence changes the inherent characteristics of 2D materials. Ion irradiation offers a very precise control over the defects with repeatable results. The defect characteristics could strongly be influenced by the ion species, ion energy, incident fluence, exposure time, and incident angle [89]. Therefore, by controlling these factors novel properties of 2D materials could be explored for developing smart electronic devices.

To achieve higher carrier mobility remains a challenge in 2D materials due to their lattice properties. There is a large gap in mobilities between theoretically predicted values and experimentally calculated values. The interfacial defects between the substrate and 2D material are one of the prominent factors in controlling the mobility of the device. The defects may occur due to the creation of a vacancy or substitution by another atom during the growth or annealing process. Chen et al. [90] have shown a mobility enhancement of 152% for monolayer MoS_2_ based transistors through strain engineering on a flat SiO_2_ substrate. Upon applying a strain of 0.87%, the effective mass of the monolayer decreases drastically boosting the carrier mobility of 2D material.

In summary, defect engineering offers a transformative approach for introducing controlled imperfections in atomically thin 2D materials. The intrinsic limitations of 2D materials could be overcome by creating intentional defects such as vacancies, dopants, edge states, grain boundaries, or interstitial atoms. The defects modified the electrical conductivity and bandgap of 2D materials through localized states in the bandgap. For chemical sensors, defects work as active sites, increasing the device sensitivity for particular analytes. For instance, sulfur vacancies in MoS_2_ make it suitable for NO_2_ detection at room temperature. Advances in defect-introducing and characterization techniques promise to enhance the scalability, reproducibility, and performance of defect-engineered 2D materials, driving innovations in nanoelectronics.

### Forming Mixed Dimensional Heterostructures

In 2D layered materials, different layered are held together by strong in-plane interaction and weak out-of-plane bonding, allowing isolation of individual layers and mixing them with other materials forming heterostructures [91]. The very visionary American physicist Richard Feynman in his very famous talk "There’s Plenty of Room at the Bottom” in 1959 asked, “What could we do with layered structures with just the right layers?” After relentless efforts over several decades, we are able to answer that question. Nowadays we are experiencing the Feynman vision, and by forming heterostructures, we are exploring those properties of materials that have never been seen in an isolated material. Recently, the vdW heterostructures obtained by stacking different 2D layered materials, have drawn huge attention due to their unique properties. These heterostructures are the basis of today’s digital electronics and smart devices possessing enormous computational power packed into a smaller space. Various characteristics such as bandgap, electron mobility, and charge carrier concentration of these heterostructures can easily be engineered according to a specific application enabling highly efficient devices. These heterostructures have already shown their potential for fabricating stretchable and flexible foldable displays, memory devices, chemical sensors, photodetectors, supercapacitors, and lithium-ion batteries.

A significant advancement in device functionality is observed by integrating 2D materials with other dimensional materials due to their dangling-bond-free surface [92]. In vdW heterojunctions, any 2D layered materials could be combined with a plethora of other dimensional materials presenting a broad class heterojunction. Hence, several kinds of new devices with exciting physics emerge by combining different dimensional materials. Such hybrids discovered exceptional electronic and optoelectronic properties of the constituent materials. The 2D materials exhibit different wave functions as compared to their conventional 3D counterparts owing to quantum confinement effects. The overlapping of atoms' electronic wave functions splits the quantized energy into discrete levels as per the Pauli exclusion principle.

**Fig. 6:**
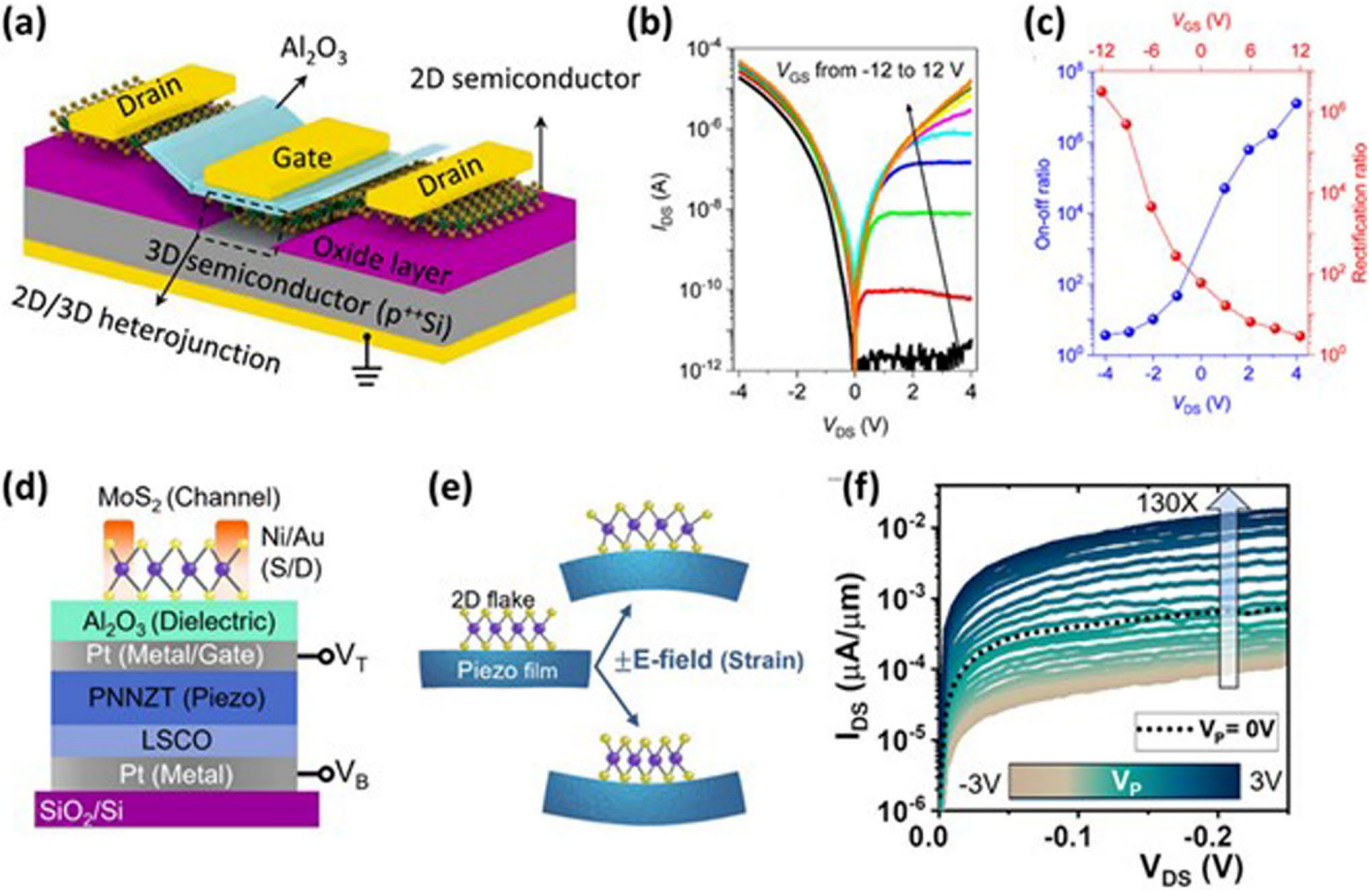
**a** Schematic of MoS_2_/Si vdW heterostructure. **b** Current–voltage characteristics of the heterojunction device upon varying gate voltage. **c** 2D/3D heterojunction diode on/off and rectification ratio as a function of drain and gate voltages, respectively. Panels **a**-**c** are reproduced with permission [93]. Copyright (2020), American Chemical Society. **d** MoS_2_ FET-based device architecture consisting of metal –piezoelectric –metal configuration. V_T_ and V_B_ at Pt electrodes indicate the voltage applied to the top and bottom electrodes, respectively. **e** Schematic representing shifting of strain from piezoelectric layer to 2D MoS_2_ via the converse piezoelectric effect. **f** Output characteristics of FET depicting improvement in drain current upon increasing the voltage difference between top and bottom electrodes. Panels **d**-**f** are reproduced with permission [94]. Copyright (2024), American Chemical Society

Among different combinations of mixed dimensional heterojunctions, 2D/2D and 2D/3D are more popular due to their versatile nature. The 2D/2D heterojunction offers precise control over the generation and transportation of charge carriers at the interface. While 2D/3D heterostructures have opened new possibilities through the integration of well-known conventional materials with emerging novel 2D materials. A high on/off ratio is required for developing high-performance logic devices. The 2D/3D vdW heterojunction showed a highly tuneable on/off ratio by moving the Fermi level in MoS_2_. The 2D/3D heterojunction between MoS_2_ and p-Si is shown in Fig. 6a [93]. The rectification ratio of the MoS_2_/Si heterojunction could be tuned by 7 orders of magnitude ranging from 0.1 to 10^6^. The output characteristics of the p–n junction diode under varying gate voltage depict strongly modulated reverse current (Fig. 6b). The on/off and rectification ratios with respect to changes in drain and gate voltages are displayed in Fig. 6c. The high performance was achieved due to the asymmetric nature of the p^++^Si/MoS_2_ heterojunction.

In recent years, mixed-dimensional heterostructures have opened new paradigms in the field of flexible and wearable electronics, particularly for healthcare applications. The 2D materials offer durability and flexibility while maintaining the high strength of the devices. In a very recent study, Varghese et al. demonstrated strain-controlled MoS_2_ heterostructure-based field-effect transistors using the piezoelectric effect [94]. The nature of transferred strain to 2D film changes from compressive to tensile by changing the biasing polarity. The device architecture consists of metal–piezoelectric–metal configuration on SiO_2_ substrate as displayed in Fig. 6d. A 2.4 nm MoS_2_ flake was exfoliated on top of the Al_2_O_3_ layer over the top electrode. The strain was transferred from a piezoelectric thin layer to 2D MoS_2_ as shown in Fig. 6e, using the converse piezoelectric effect. As the potential difference between the top and bottom electrodes increases from 0 to ± 150 kV cm^−1^, the drain current could be modulated nearly 130 times, as depicted in Fig. 6f.

Gibbs and Helmholtz's free energy of 2D materials indicates their thermodynamic stability and their integration with other dimensional materials [95]. For instance, the CVD and PVD growth of materials requires minimizing free energy in the system. In mixed-dimensional heterostructures, free energy also controls the interactions and the adhesion between 2D materials and other dimensional materials. The uniform stacking of different layers of 2D materials through vdW interaction without any chemical bond is also governed by free energy. The reduction of interfacial free energy facilitates efficient charge transfer in FETs, photodetectors, and quantum devices. The Gibbs free energy establishes the charge storage and electrochemical interactions in supercapacitors. Similarly, the Helmholtz free energy controls the conversion efficiency in energy harvesting devices. In chemical sensing and environmental monitoring applications requiring functionalizing of 2D materials, the systems are designed to minimize free energy for improved selectivity and sensitivity for target analytes.

Enthalpy indicates the total energy in a system impacting the engineering technique for developing 2D materials-based smart devices. Enthalpy controls the stability of 2D materials influencing their integration to form heterostructures [96]. For example, the CVD growth of 2D material involves exothermic reactions where enthalpy changes ensure the synthesis of defect-free layers. In vdW heterostructures, interlayer interactions, stability, and layer alignment depend on enthalpic contributions. Thermal management in smart devices also depends on enthalpy. Heat dissipation in compact devices is deeply tied to enthalpic interactions and enthalpy changes. In supercapacitors and batteries, the charge storage capacity strongly depends on enthalpy changes during ion adsorption and desorption processes. Therefore, smart devices with superior functionality and reliability could be designed by optimizing enthalpy-related processes.

Therefore, the synergistic effects of mixed-dimensional heterostructures offer a ground-breaking approach to enhancing the functionalities of 2D materials-based smart devices. The selection of constituent materials in heterostructures to meet the specific application depends on computational modeling and theoretical predictions. The unattainable properties in individual materials could be obtained by combining 2D materials with 0D, 1D, or 3D components. By integrating 2D materials with other suitable materials, carrier dynamics and band alignment at the heterointerfaces could precisely be tuned for particular applications such as tunnelling FETs and photodetectors. In addition, the heterostructures improve the light-matter interactions and broaden the spectral response, enabling the development of highly efficient optical devices.

## Selection of 2D Materials for Smart Devices

**Fig. 7:**
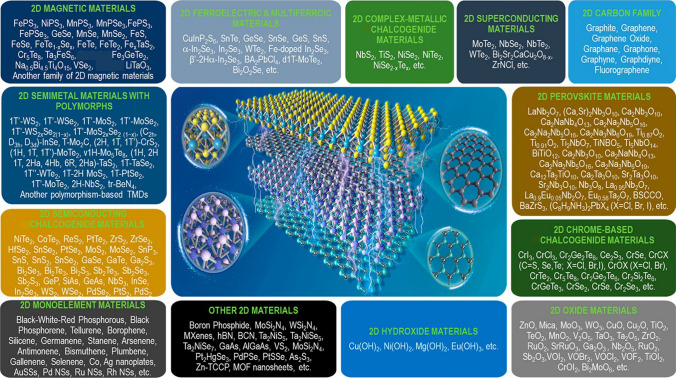
Classification of the 2D materials depending on their diverse properties. Reproduced with permission [97]. Copyright (2022), American Chemical Society

Since the discovery of graphene (the first 2D material) at The University of Manchester in 2004, a plethora of materials has been added to the family of 2D materials [53]. Although they share similar types of structures, incredibly diverse properties of the 2D materials help to include metals, semimetals, semiconductors, and insulators in their family. The numerous 2D materials are classified with their diverse properties, as shown in Fig. 7 [97]. The selection of the 2D material for device manufacturing is crucial and that depends on the specific material properties required for a particular application. For example, although graphene as a first 2D material has remarkable electrical, optical and mechanical properties, it is not suitable as channel of digital transistor device owing to its zero-band gap. However, some research efforts have been made to open band gap in the graphene through designing new structure including nanoribbon, nanowire, nanotube, and bilayer graphene. The graphene nanostructures are still facing challenge to achieve ideal performance of the transistor due to decreased carrier mobility and subthreshold swing. Moreover, large specific surface area, chemical stability, high thermal conductivity, huge strength and excellent optical properties of the graphene makes it promising candidate for water purification, sensing, hydrogen production, antimetastatic agent and optical applications. In addition, discovery of Ti_3_C_2_ in 2011 has introduced dozens of 2D transition metal carbides, and nitrides (known as MXenes) materials. Metallic layered structures, high mobility, rich surface chemistry, and hydrophilicity properties of the MXenes are suitable for energy storage device applications. On the other hand, the surface of the 2D materials without dangling bonds supports synthesizing vdW heterostructures without lattice mismatching. These distinctive vdW heterostructures offer new physics and unique functionality for developing smart devices [98–100]. However, selection of the 2D materials is very crucial in the synthesis of the vdW heterostructures. Thermal and chemical stability of the materials, and kinetics and thermodynamic growth process are some of the considerable parameters for the synthesis of vdW heterostructures using the semiconductor industry compatible CVD process. The graphene or hBN cannot be grown over the 2D TMDs because of its higher temperature growth. Moreover, growth of the 2D MoS_2_ is possible over the 2D WSe_2_ surface without exchange of the S/Se atoms. However, 2D WSe_2_ could not be grown on the 2D MoS_2_ film without exchange of the S/Se atoms because sulfur vacancies would be created, and subsequently occupied with Se during the high temperature growth of the WSe_2_.

In the context of manufacturing, synthesis of the 2D materials at the industry level is compatible with conventional Si planar technology because these materials have large (macroscopic) lateral dimensions despite having only nanoscopic thickness (vertical dimension) [101]. Moreover, the easy transfer of the processed 2D materials from one platform to another makes them suitable to integrate with the present technologies, especially, at BEOL in the CMOS technology process [102]. We believe that 2D materials represent strong material systems for developing smart computational as well as non-computational electronic devices. In the context of computational, the field effect transistor (FET) is a fundamental electronic device. The operation of the FET in digital as a digital switch (on and off) inspires the selection of semiconductor 2D materials so that the conductivity of the material can be easily modulated by the external applied voltage. From this view, layered TMDs materials with the general formula MX_2_, where M and X are transition metal and chalcogen elements, respectively, are more explored, and semiconducting TMDs such as MoS_2_, WS_2_, and WSe_2_ have shown huge potential in the field of the computational device [103]. The 2D TMDs semiconductor materials have been utilized as a transistor channel material. These materials also fulfill the current demand for advanced technology nodes by fabricating FET devices of sub-10 nm channel length. For example, 2D MoS_2_ as a channel material is used to fabricate an FET device of 1 nm channel length [20]. On the other hand, a high-k dielectric gate material is also required to give sufficient electrostatic control with minimal gate leakage current. Moreover, it should preserve the intrinsic properties of the channel 2D material. 2D hexagonal boron nitride (h-BN) as an insulating material is widely used as a gate oxide material for developing high-performance 2D material-based devices. However, its good electrostatic controlling behavior is limited to its single or double layers, and beyond these layers, a high gate leakage current is observed. The 2D channel materials have large contact resistance and the high contact resistance limits the benefits of channel materials. The dominance of contact resistance over the channel conductivity severely affects the performance of the device. So, low Ohmic contact resistance schemes are essential for improving the performance of the device in an IC chip. Overall, an innovative approach to contact and interface engineering, and suitable dielectric materials are urgent needs for the 2D materials integration in computing devices [104]. Besides the use of the 2D materials in computational systems, these are also employed in various non-computing devices including gas or chemical sensors, biosensors, photodetectors, image sensors, superconductors, radio-frequency devices, magnetic devices, thermoelectric and piezoelectric devices [105–112]. These vast applications of 2D materials are because of their remarkable diverse properties. Inherent high surface-to-volume and versatile functionality of the 2D materials are suited for gas/chemical sensor and biosensor applications [113].

## Device Architectures

### Field-Effect Transistors

**Fig. 8:**
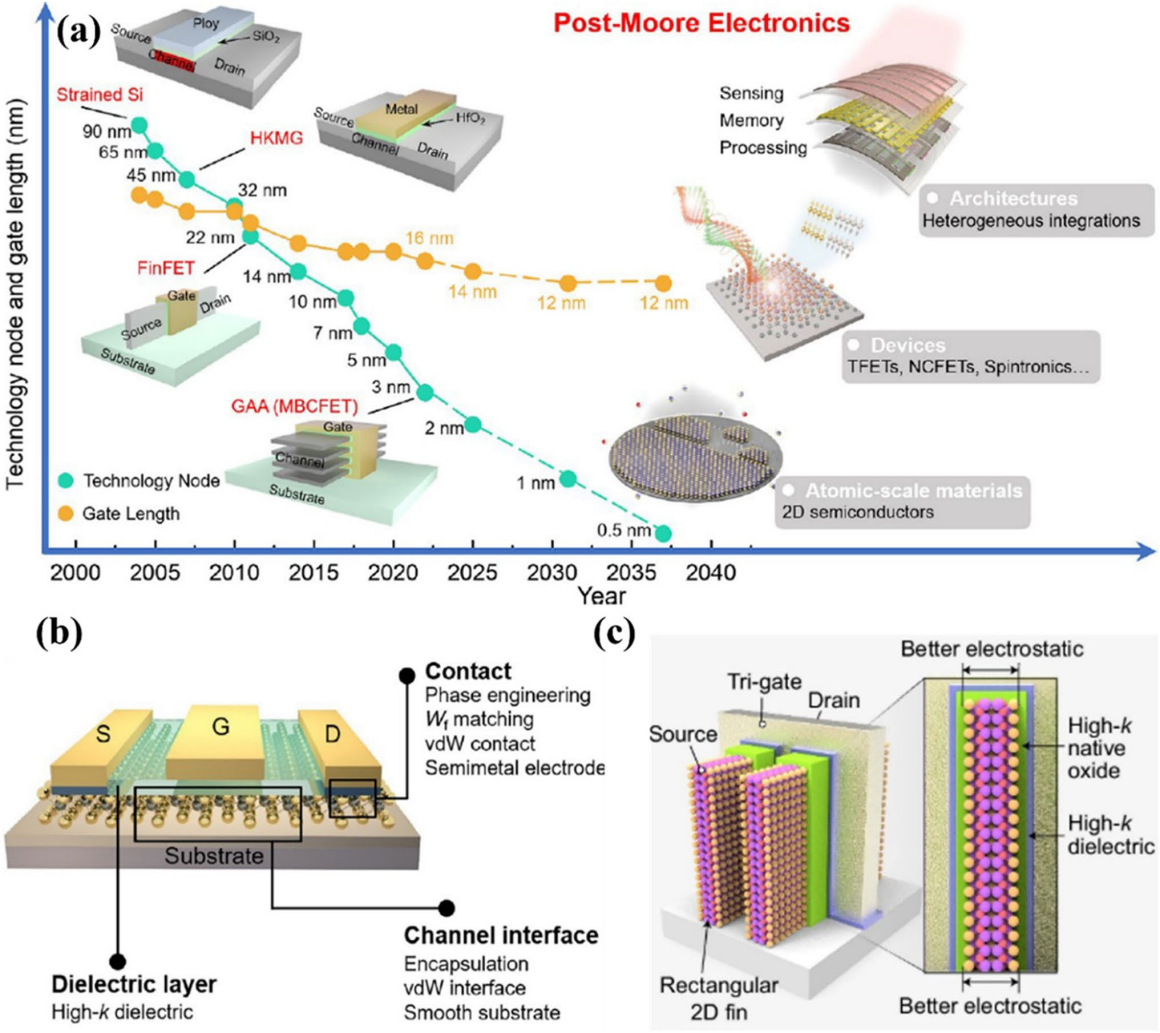
**a** Scaling of CMOS transistors and further scaling, post-Moore electronics, would be based on 2D materials and unconventional devices. **b** Three approaches including contact electrode, channel interface, and dielectric layer are essential to optimize the performance of 2D materials-based transistors. Panels **a** and **b** are reproduced with permission [104]. Copyright (2024), American Chemical Society. **c** Schematic illustration of the 2D multi-fin FET structure. Reproduced with permission [120]. Copyright (2024), Springer Nature

Field effect transistor (FET) is the most important electronic device in the electronic industry, and it is a basic building block of modern ICs. The scaling of the transistor’s size has become a continuous process to follow Moore’s law for improving the performance, miniaturization, and cost of the ICs. The evolution of transistors, scaling, and post-Moore electronics are shown in Fig. 8a [104]. The further scaling depends on the emerging 2D materials, unconventional devices, and innovative architectures. Nonetheless, the scaling rate has slowed in the last few years because of achieving the threshold limit of the thickness of the conventional 3D Si material. The thickness of the semiconductor and dielectric play a crucial role in defining the characteristic length of the conventional nonplanar fin field-effect transistor (FinFET) structure. The characteristic length is defined as (*λ* ≈ *t*_channel_ + (*ε*_channel_/*ε*_oxide_)*t*_oxide_) [114]. It suggests that the thickness of channel material and dielectric should be less. The reduction of the thickness of 3D Si below 5 nm severely deteriorates the mobility at the interface of Si and dielectric through surface scattering [115]. The high degradation in mobility limits the further scaling of the transistor. Conversely, atomically thick 2D semiconductor materials (thickness < 1 nm) manifest huge potential to further scaling beyond the limit of 3D Si material. The mobility (~ 100 cm^2^ V^−1^ s^−1^) of the 2D MoS_2_ (thickness ~ 0.65 nm) is much higher than the sub-5 nm thickness of the 3D Si. The 2D MoS_2_ also exhibited high ‘on’ currents of > 400 μA μm^−1^ and a good subthreshold slope of 80 mV decade^−1^ with a channel length of 10 nm [116]. The transistor of 1 nm channel length was also fabricated using the 2D MoS_2_ as a channel and a carbon nanotube as the gate electrode [20]. Furthermore, a FET transistor fabricated with 2D black phosphorus showed a higher ‘on’ current of 1 mA μm^−1^ because of its low bandgap and high carrier mobility [117]. Many efforts have been made to integrate 2D materials in FET to design n-channel, p-channel, and ambipolar transport by exploiting different semiconductors, contact, and doping. Despite the advancement in 2D FET, the non-ohmic behavior of contacts is still a challenge for the 2D channel material. Recently, an ultralow contact resistance of 42 Ω μm was achieved using semimetal antimony (Sb) contact for the monolayer MoS_2_ [118]. The properties of the 2D materials are highly dependent on the quality of surface and interface because of their atom level thickness. Therefore, suitable dielectric 2D materials, contact, and interface engineering help to improve the performance of the 2D materials-based transistor devices (Fig. 8b) [104]. In addition, many unconventional performance-enhancing technologies such as negative capacitance, floating-gate transistors, tunneling transistors, and ferroelectric transistors have been exploited with the 2D channel material [119]. Beyond the charge-based transport in complementary metal–oxide–semiconductor (CMOS) technology, 2D materials are also utilized in spin transistors and excitonic transistors. These innovative approaches would provide thrust to applying 2D materials in post-Moore ICs.

For the planar FinFET structure, further reducing the modern industry node below 5 nm degrade performance severely and also creates issues in the fabrication process because of the limitation of fin width and fin pitch of the modern FinFET structure. So, semiconductor industries are now shifting from the trigate FinFET structure to gate-all-around field-effect transistors (GAAFETs) for achieving high electrostatics control by the increased gate, and that will lead to a paradigm shift in transistor technology [121, 122]. On the other hand, recently, Yu et al. reported a multi-fin FET structure using the 2D Bi_2_O_2_Se fin arrays with high-k native-oxide Bi_2_SeO_5_, as shown in Fig. 8c [120]. The multi-fin FET device exhibited an excellent on/off current ratio (˃ 10^6^) with high durability. The multi-fin FET provides higher drive current, transconductance, lower noise, and higher integration density than the single-fin FET in the 2D electronics logic devices.

### Complementary Metal–Oxide–Semiconductor

**Fig. 9:**
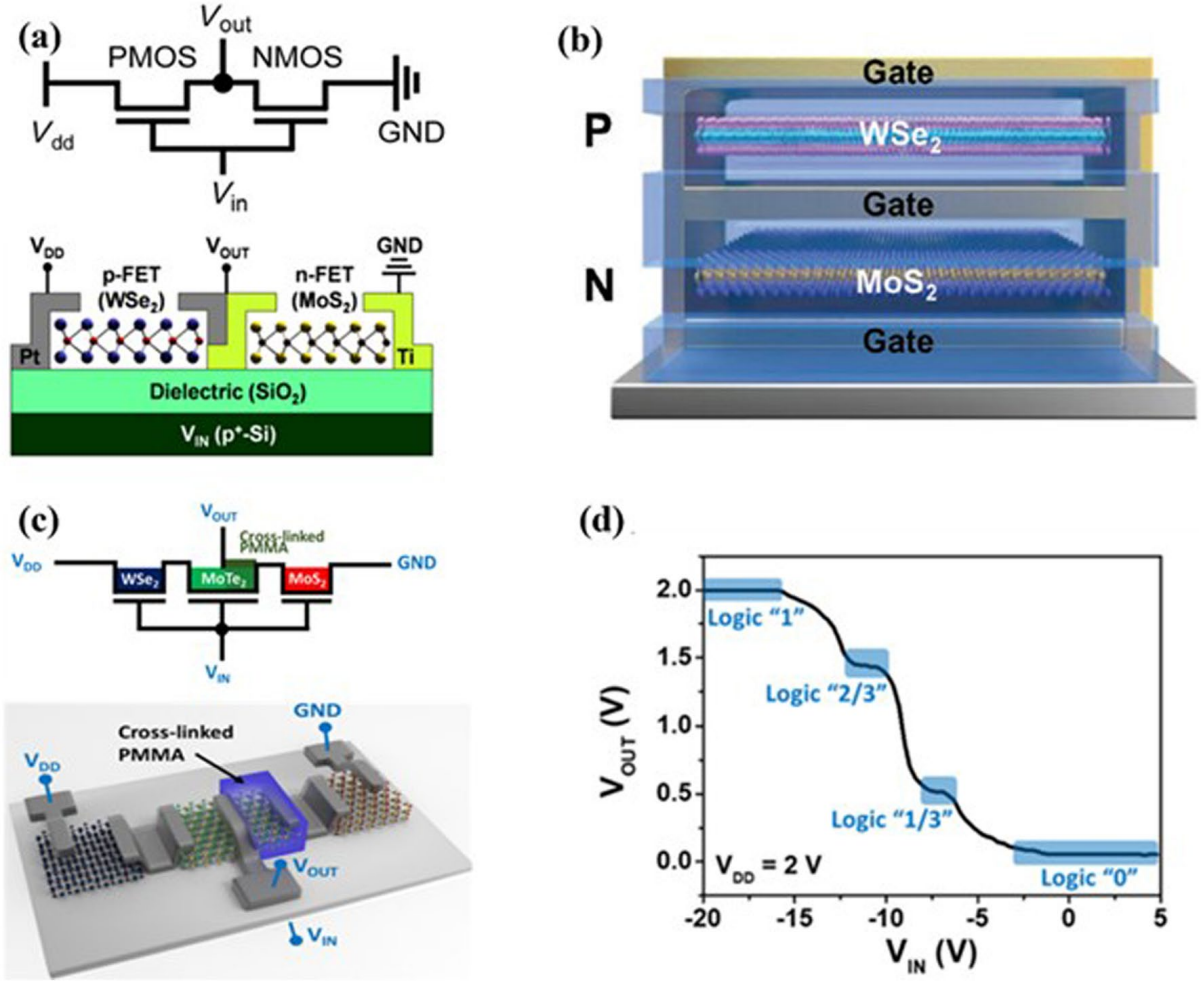
**a** Circuit diagram and device structure of CMOS inverter consisting of connected n-FET of MoS_2_ and p-FET of WSe_2_ in series. Reproduced with permission [123]. Copyright (2015), American Chemical Society. **b** Device structure of the complementary field-effect transistors (CFET) with top p-type WSe_2_ FET and bottom n-type MoS_2_. Reproduced with permission [124]. Copyright (2022), Wiley–VCH. **c** Circuit diagram and quaternary inverter device consisting of a WSe_2_/MoTe_2_ heterotransistor in series with a PMMA –MoTe_2_/MoS_2_ heterotransistor. **d** Four distinct logic states: “1”, “2/3”, “1/3”, and “0” of the quaternary inverter. Panels **c** and **d** are reproduced with permission [125]. Copyright (2021), American Chemical Society

A complimentary metal–oxide–semiconductor (CMOS) logic device is used in the development of state-of-the-art digital integrated circuits (ICs) chips or microprocessors which is the backbone of modern computing and system design. The CMOS is made of two connecting complementary FETs: a PMOS and an NMOS in series. In the operation of the CMOS, one transistor is off, and another is on condition at the same time, which reduces the static power of the logic device. For example, an applied low input voltage turns on the PMOS and off the NMOS resulting in a high output voltage (~ *V*_dd_). In contrast, an applied high input voltage turns on the NMOS and turns off the PMOS resulting in low voltage (~ 0). Despite the individual transistor logic device, the CMOS logic device exhibits low power consumption through reducing static power consumption and high noise tolerance. A 2D CMOS inverter device was fabricated by connecting two separate p-type 2D WSe_2_ and n-type 2D MoS_2_ devices in series using the direct imprint method, as shown in Fig. 9a [123]. The 2D hetero-CMOS inverter device exhibits good performance: maximum voltage gains of ∼27, dynamic switching of ∼800 μs, noise margin of 0.5*V*_DD_ (supply voltage, *V*_DD_ = 5 V) with a transition voltage of 2.3 V, and very low power consumption in the range of sub-nano watt. Good gate patterning and isolation between p- and n-type wells are supported to reduce overlap capacitance values. Moreover, thinner flakes and encapsulation of the device helped to address input –output signal mismatch (caused by negative transition voltage in the CMOS inverter). On the other hand, the leading microelectronics industries would still be using the conventional Si-CMOS technology by the vertical stacking of the p- and n-type FET devices as represented to 3D monolithic CMOS integration, despite conventional two planar transistors connected in series. There is no doubt that the multibridge complementary field-effect transistors (MBCFET), and complementary field-effect transistors (CFETs) architectures with vertical stacking of n-type and p-type FETs lead to scaling by reducing the layout area. However, further scaling the CFETs for future node technology, and replacing Si would be essential from the atomically thick material with high mobility and low leakage current. The atomically thick 2D semiconductor TMDs materials have huge potential to be an integral part of the CFET. Liu et al. fabricated CFETs with vertical stacking of n-type and p-type FETs using the monolayer of MoS_2_ and WSe_2_, respectively [124]. The CFET device is shown in Fig. 9b. The CFET with channel materials thickness < 1 nm showed good performance and that provided a technological base for new transistor technology. In addition to binary logic CMOS devices, multivalued logic CMOS devices based on 2D materials are getting more attention because these devices reduce the power consumption and integration complexity of the ICs. Moreover, the use of the multivalued logic CMOS in the IC chip would improve heat dissipation by reducing the number of interconnected lines and devices. In this context, Son et al. reported a multivalued ternary logic device using p-type WSe_2_, n-type MoS_2_, and ambipolar MoTe_2_ transistors connected in series [125]. They further partially functionalized the ambipolar MoTe_2_ with a cross-linked poly (methyl methacrylate) (PMMA) layer to develop quaternary inverter through specially controlled n-type doping of the MoTe_2_, as shown in Fig. 9c. The logic device showed four stable logic states as 1, 2/3, 1/3, and 0 for applied supply voltage *V*_DD_ = 2 V, and corresponding *V*_in_ and *V*_out_ voltages to all four logics are shown in Fig. 9d. Although lots of advancements have been demonstrated in the CMOS logic devices using the 2D materials, several issues including doping, scalability and uniformity in large area 2D materials growth are still creating hurdles to implementation of the 2D materials in the logic circuits design for commercial applications.

On the other hand, integration of the 2D transistor with the conventional 3D Si-CMOS technology is also a hot research interest worldwide rather than completely replacing the Si from the 2D materials [101]. The integration of the 2D materials with the Si chip forms a three-dimensional monolithic construction that provides a heterogeneous platform for improving the performance as well as functionality of the Si chip [102]. The incorporation of 2D materials into monolithically integrated Si-chips helps to improve packaging through increasing devices per unit area and integration density, and that leads to the “More Moore” technology through facilitating scaling and maximum area utilization [126]. For example, exploitation of doped-graphene-nanoribbon in interconnect has been reduced by more than 50% interconnect thickness with lower parasitic, power consumption, and interconnect delays [127]. On the other hand, “More than Moore” technologies are also supported by improving the multifunctionality of the Si-chip after integrating non-computation devices including sensor, and memory in different tiers of the third dimension. In addition, Goossens et al. developed a broadband image sensor after transferring graphene onto a Si-CMOS chip as monolithic 3D heterogeneous integration of 2D materials with silicon [128]. The deposition of PbS colloidal quantum dots (as the light absorption layer) onto the patterned graphene pixels supported to achieve high gain and photoresponsivity through exploiting photogating effect and fast charge transfer from the quantum dots to the graphene. The 2D graphene integration with 3D Si-chip improved the responsivity and broadband range of 300–2,000 nm owing to remarkable optical and electrical properties of the graphene. Moreover, optical properties of the optical devices could be tunned electrically with integration of graphene with devices through remarkable graphene Dirac fermion tuning. In this regard, Yao et al. integrated graphene with optical devices and develop electrically controlled tunable frequency combs in graphene–nitride microresonators and laser frequency combs in graphene-fiber microresonators [129, 130].We believe that 2D materials can be one of the most suitable materials for the construction of the 3D monolithic IC with advanced functionality. However, some feasible fabrication processes and semiconductor infrastructure are still required for the direct growth of the 2D materials on the Si chip from the industry perspective.

### Memory

**Fig. 10:**
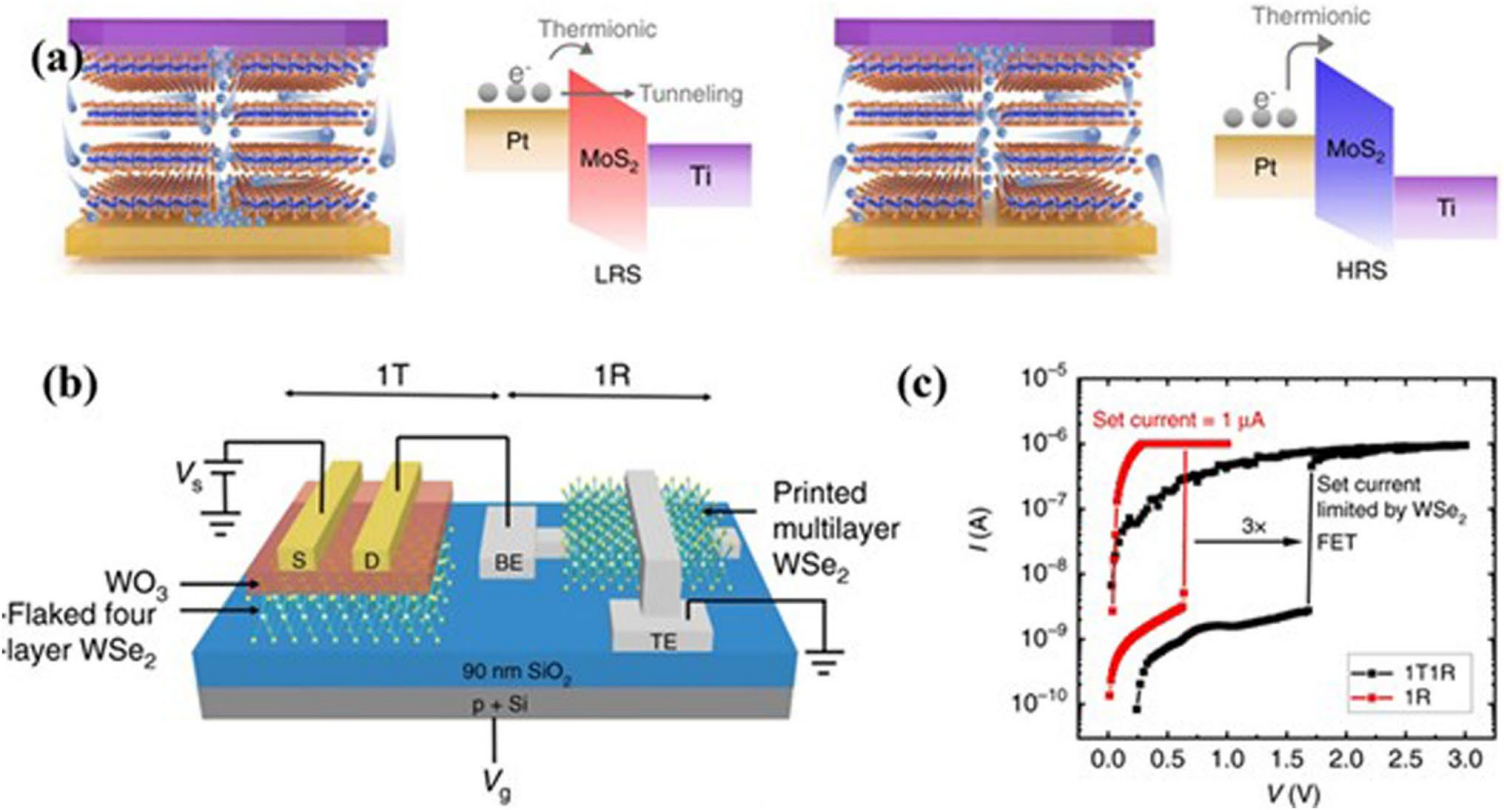
**a** Resistive sensing mechanism of the 2D MoS_2_ based on the sulfur vacancy: set and reset process and corresponding energy band diagram of the device. Reproduced with permission [133]. Copyright (2022), Springer Nature. **b** Device structure of the 1 transistor and 1 memory cell using the 2D WSe_2_. **c** I-V switching diagram of the 1T1R device configuration. Panels **c** and **d** are reproduced with permission [134]. Copyright (2019), Springer Nature

In the era of the Internet of Things, AI and big data, memory is a leading electronic device to process and store data. Nowadays, flash memory is mostly used in smart consumer devices including smartphones. In conventional computational architecture, data is transferred from off-chip memory storage to an on-chip computation unit and that results in high latency and high energy consumption. So, scientific and technical innovations in solid-state memories are required to provide efficient computation and data storage on the same chip. From this view, many research efforts have been made to innovate memory devices to fulfill current and future demands of the microelectronics industry. In the context of nonvolatile memory, several innovative technologies including resistive random-access memory (RRAM), magnetoresistive random-access memory (MRAM), and phase-change memory (PCM) have been progressively developed [131]. The 2D materials and their vdW heterostructures have shown huge potential to develop these memories for making useful because of their excellent electronic properties and low thermal budget [131]. The 2D graphene is utilized as a planar electrode in the development of scalable vertical RRAM. The integration of 3 Å thin graphene into RRAM reduces the vertical height and cost of the 3D vertical stacked memory structure. In regards to ferroelectric memory, several 2D materials such as graphene, TMDs and Black phosphorous have been exploited into FeFET and these materials help to prevent the migration of atoms from ferroelectric material into the transistor channel. That leads to interface stability for storing binary states through the direction of spontaneous electric polarization. Despite the joule heating in the PCM, the PCM shows good endurance and a feasible lifetime of ˃10^9^ cycles. To further reduce the power consumption, graphene as a thermal barrier was employed in between the phase change materials. The graphene reduced the 40% RESET current of the PCM because low out-of-plane thermal conductivity of the graphene helps to confine the generated heat inside the device. Besides the good performance of in conventional memory systems, 2D materials have also shown its potential in the novel a metal–insulator–metal (MIM) memory structure. Ge et al. reported first-time nonvolatile resistance switching in single layer of the TMDs (MoS_2_, MoSe_2_, WS_2_, WSe_2_) materials [132]. Atomically thin TMDs have sharp interfaces and clean tunnel barriers, which support to prevention of excessive leakage stored charges in the floating gate. They offer low programming voltage, good stability, and high integration density. Zheng et al. reported a phase change RRAM using the 2D TMDs (vertical 2H-MoTe_2_- and Mo_1−*x*_W_*x*_Te_2_) [59]. An electric field was exploited to change the phase of the MoTe_2_- and Mo_1−*x*_W_*x*_Te_2_. The devices exhibited good resistive switching by changing states between high and low resistive within 10 ns. Moreover, different defects including grain boundary, edge, and vacancy defects of the 2D TMDs materials also exploited for nonvolatile resistive switching. Tang et al. reported that interflake diffusion of sulfur vacancy through the edges of the 2D MoS_2_ supports forms a conductive filament in the device, as shown in Fig. 10a [133]. The percolation of the sulfur vacancies would depend on the size of the flakes. The electrons would reach Ti from the Pt electrode through the formed sulfur vacancy filament in the MoS_2_ and that results in a low resistance state. The device would be reset after rupturing the filaments by changing the polarity of the Pt electrode. Besides the development of ultrafast and ultra-thin single memory devices, 2D materials are also used to fabricate transistor selectors in memory arrays. That helps to reduce leakage current. The sneak current in the RRAM array is responsible for increasing crosstalk and static power consumption. From this view, Sivan et al. proposed 1 transistor 1 resistive memory (1T1R) cell using the 2D WSe_2_, as shown in Fig. 10b [134]. The 2D WSe_2_ thin film transistor (TFT) did successfully resistive switching in the WSe_2_ RRAM (Fig. 10c).

### Tiered Framework

Tiered Framework, combining multiple materials or structures, addresses challenges like restacking in 2D nanosheets while leveraging synergistic effects among components. This approach increases specific surface area and enhances electrode–electrolyte wettability, promoting redox reactions and ion transport for high-performance micro-supercapacitors.

**Fig. 11:**
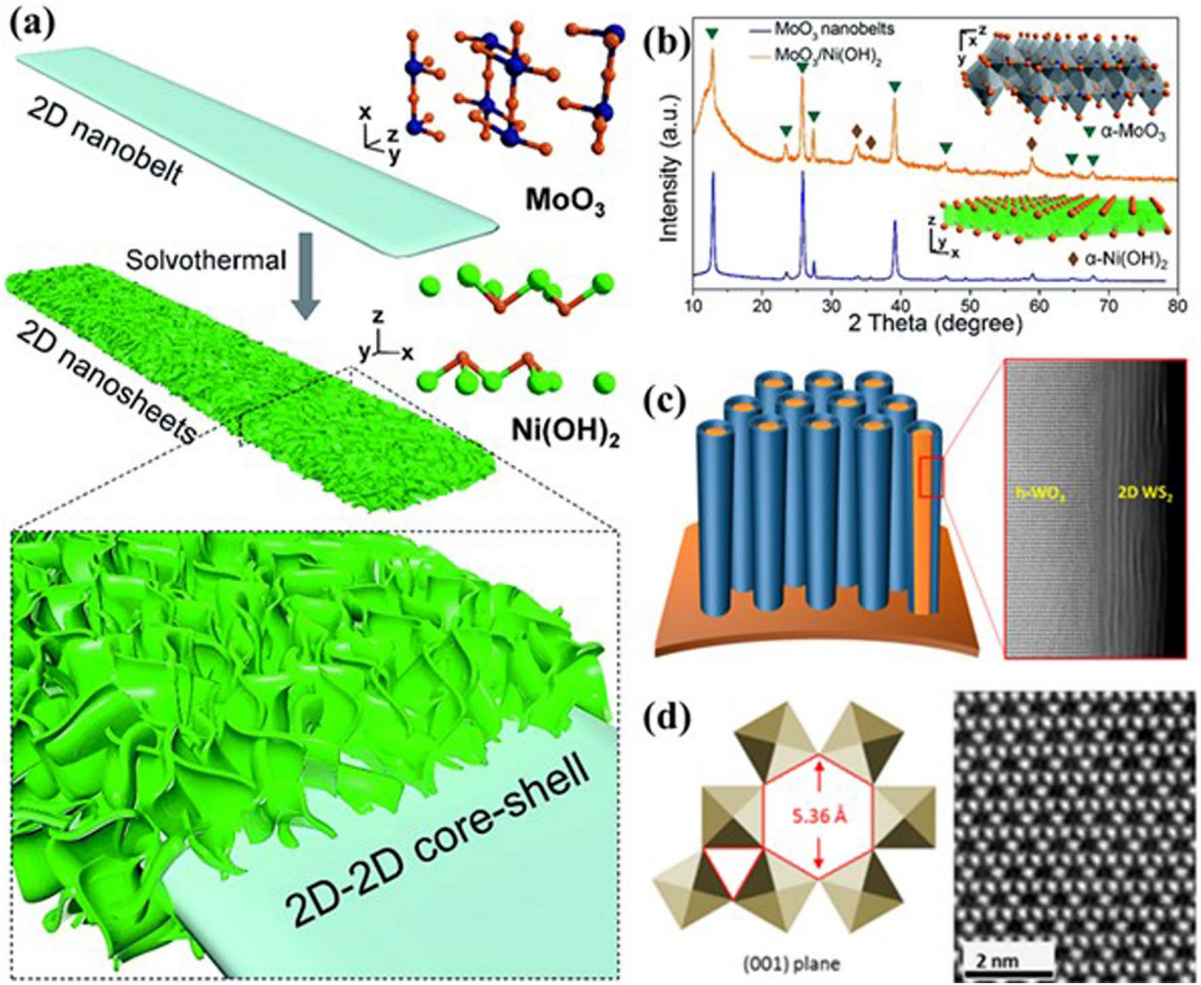
**a** Schematic illustration of hierarchical 2D-2D MoO_3_/Ni(OH)_2_ manufacturing process. **b** XRD spectra displaying the MoO_3_/Ni(OH)_2_ combination and MoO_3_ nanobelt diffraction patterns. Upper and lower insets show the crystalline forms of α-MoO_3_ and α-Ni(OH)_2_, respectively. Panels **a** and **b** are reproduced with permission [135]. Copyright (2017), Royal Society of Chemistry. **c** A schematic representation of a one-body arrangement of core/shell nanowires with 2D-WS_2_ and WO_3_ layers. **d** Illustrations of crystalline structures showing the h-WO_3_ nanowire's architecture along with accompanying ADF-STEM pictures. Within the (001) basal plane, the cross-sectional view shows the nanowire's robust framework. Panels **c** and **d** are reproduced with permission [136]. Copyright (2016), American Chemical Society

Improved functional performance was achieved via a hybrid composite formed by mixing MoO_3_ nanobelts and Ni(OH)_2_ nanosheets, as described by Zhu et al. [135] in their solution-processed 2D-2D hierarchical design. To improve mass transport and surface interactions, 2D nanosheets possess substantial surface area have been coupled with a thin 2D nanobelt core. The highly anisotropic orthorhombic α-MoO_3_ was fabricated using hydrothermal and solvothermal methods, confirmed by XRD and TEM analyses (Fig. 11a, b). The crystalline structure of Ni(OH)_2_ was verified by the enhanced XRD peaks corresponding to (110) and (300) planes (Fig. 11b insets). The resulting 3D hierarchical architecture provides short ion diffusion pathways, delivering bifunctional electrochromism and supercapacitive properties. This system is well-suited for use in smart buildings, cars, and electronics because to its high specific capacitance, pseudocapacitive storage, coloring efficiency, and electrochromic optical modulation. Building upon the concept of hierarchical structures, researchers have explored the integration of 2D materials to further enhance performance. Supercapacitors made of 2D TMDs have a lot of potential because of their layered architectures and significant surface areas. However, some drawbacks such as high capacitance loss and limited cycle stability remain present due to random assembly of materials. Figure 11c shows the highly efficient core/shell nanowire supercapacitors developed by Choudhary et al. [136] that incorporate one-dimensional (1D) h-WO_3_ nanowires with conformal 2D WS₂ layers. Durable and atomically sharp core/shell contacts are guaranteed by this "one-body" arrangement, which is accomplished by successively oxidizing and sulfurizing identical metal current collectors. The h-WO₃ core (Fig. 11d) comprises W–O₆ octahedra arranged along the [001] zone axis, forming open hexagonal channels (~ 5.36 Å diagonal) confirmed by ADF-STEM imaging. These hybrid structures synergize the benefits of 1D and 2D components, offering high surface area, mechanical robustness, and distinct functionalities, resulting in exceptional capacitive performance and over 30,000 charge–discharge cycles with minimal degradation. Another significant advancement in 2D material-based energy storage devices involves the understanding of surface redox charge storage mechanisms in hierarchical heterostructures. In addition, Mahmood et al. [137] used Raman spectroscopy and in situ synchrotron X-ray absorption to study the distinct surface redox charge preservation process in 3D hierarchical hybrid structures made of WS_2_ layer. The combination of 3D connectivity with heteronanosheets results in extremely reversible and rapid capacitive performance in these 3D structures that integrate 2D-WS_2_ heteronanosheets. Due of distinct lattice vibration modes, compositionally similar 2D-WS_2_ structures function very differently from this. They do not have the same quick and high capacitance. These 3D hybrid structures have better capacitive performance because protons move quickly into the in-plane W-S lattice framework, surface-exposed intralayers go through reversible redox alterations within the electrochemically engaged 1T phase, and the oxidation state changes when protons enter and leave the structure. This is shown by the E_2g_ bands becoming softer and the W-W bond attributes alterations.

In consequence, hierarchical structuration of low-dimensional materials, from 2D-2D architectures to 3D hierarchical heterostructures, has demonstrated significant advancements in energy storage technologies. The integration of materials addresses challenges such as restacking, poor interface integrity, and limited cyclic stability, while enhancing surface area, ion transport, and redox activity. These innovative designs not only achieve exceptional capacitive performance and long-term stability but also provide valuable insights into surface-dominant charge storage mechanisms. Collectively, these approaches highlight the immense potential of engineered multidimensional materials for next-generation energy storage applications.

## Applications of the Smart Devices

### Flexible and Wearable Electronics

**Fig. 12:**
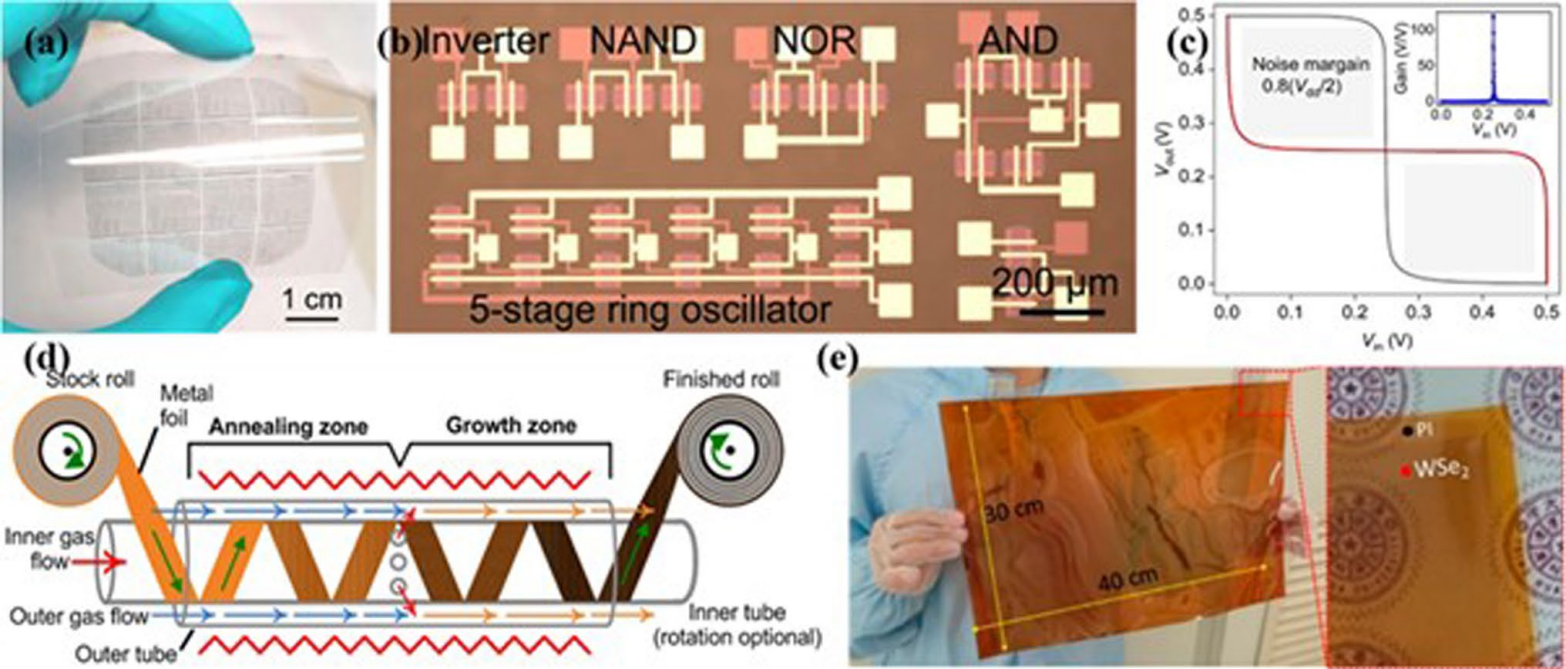
**a** Image of 2D MoS_2_ TFT on flexible (4 × 4 cm^2^) PET substrate. **b** Optical image of 2D MoS_2_ based inverter, NAND, NOR, AND, and ring oscillator. **c** Voltage transfer curve of the MoS_2_ inverter. Panels **a**-**c** are reproduced with permission [81]. Copyright (2023), Springer Nature. **d** Schematic of concentric tube CVD system configured for R2R graphene growth. Reproduced with permission [141]. Copyright (2015), Springer Nature. **e** Photograph of the CVD-grown WSe_2_ on a large flexible PI substrate (30 × 40 cm^2^). Reproduced with permission [142]. Copyright (2017), American Chemical Society

Nowadays, the development of flexible and wearable electronic devices is an emerging research area, and these devices play an integral role in different applications, such as information technology, energy generation and storage, sensing, and healthcare. Conventional flexible electronic devices are widely fabricated using organic semiconductors, amorphous and polycrystalline forms of silicon, and metal oxide materials. In the past few years, 2D materials have been more studied in flexible and wearable electronics owing to their excellent electrical and mechanical properties [138–140]. Their ultimate thickness controllability, integration flexibility, and compatibility with conventional CMOS technology make them promising materials for developing smart electronic devices on flexible substrates [7]. Graphene with inherent zero band gap is limited to employ in logic devices as a channel, but it has been more explored as an electrode in optoelectronic devices, sensors, and non-transistor applications. Moreover, it is explored for developing different RF circuits because of its high mobility and saturation velocity with an inherent ambipolar nature. Other 2D materials such as semiconducting TMDs and phosphorene are suitable for logic devices owing to their tunable band gap [138]. Tang et al. fabricated a low-power and high-performance flexible IC using the 2D MoS_2_ and that includes essential building blocks of ICs such as inverters, NAND, NOR, AND gates, and ring oscillators, as shown in Fig. 12a, b [81]. The inverter showed a good noise margin with a high voltage gain of 397 and low power consumption of 10.3 pW μm^−1^ at *V*_dd_ = 1 V. The voltage transfer curve of the 2D MoS_2_ inverter is shown in Fig. 12c. The ring oscillator exhibited a low propagation delay. The higher performance of the flexible IC suggests that 2D MoS_2_ is a promising channel material for transistor technology for low-power and high-performance applications. Several direct band gap 2D materials are also explored in flexible optoelectronics devices and show good optical properties.

Large phonon energies, high dielectric breakdown field, and high in-plane thermal conductivity of the insulating 2D h-BN support to improve 2D charge transport in 2D materials-based transistors. 2D h-BN with good mechanical properties proves a good dielectric gate material for flexible electronics. Moreover, the suitable thickness of the 2D h-BN acts as a good thermal management system for plastic substrates by spreading heat to in-plane metal electrodes and isolating heat out-of-plane owing to its anisotropic thermal properties. Thermal management is more crucial for the 2D materials-based flexible device because high current density with fast transport may lead to an increase in the peak temperature. That increased temperature would be more than the glass transition temperature of plastic substrates. Besides the flexibility of the device, stretchability, biocompatibility, and high bending angle are also required for conformal electronics, bioelectronics, and body-mounted devices. The elastic limits of the 2D materials with minimum grain boundaries are much better than conventional 3D semiconductors. The higher elastic limit and atomically thin thickness of the 2D materials offer more advantages in the flexible and wearable electronics field than the conventional 3D semiconductors. On the other hand, large-area nanofabrication of the 2D materials and device fabrication on flexible substrates are prerequisites for flexible and wearable electronics. In this regard, many innovative methods (roll-to-roll (R2R) process for graphene growth, Fig. 12d) have been used for large area growth of the 2D materials [141]. Typically, the 2D materials are first grown on the rigid substrates and then transferred on the soft substrates by using different extra transfer methods. These post-growth transfer techniques degrade film quality by adding wrinkles, defects, and a residue of the polymer during the transfer process. To address this issue, some significant research efforts have been made to directly grow 2D materials on the flexible substrate using a low-temperature plasma CVD process. Medina et al. used an inductively coupled plasma CVD process and a pre-deposited WO_3_ film on a flexible PI substrate was selenized at a low temperature of 250 °C through decomposed high-energy selenium ions [142]. A large area high quality monolayer WSe_2_ grown on large flexible substrate (area = 30 × 40 cm^2^) (Fig. 12e).

### Biomedical Applications

**Fig. 13:**
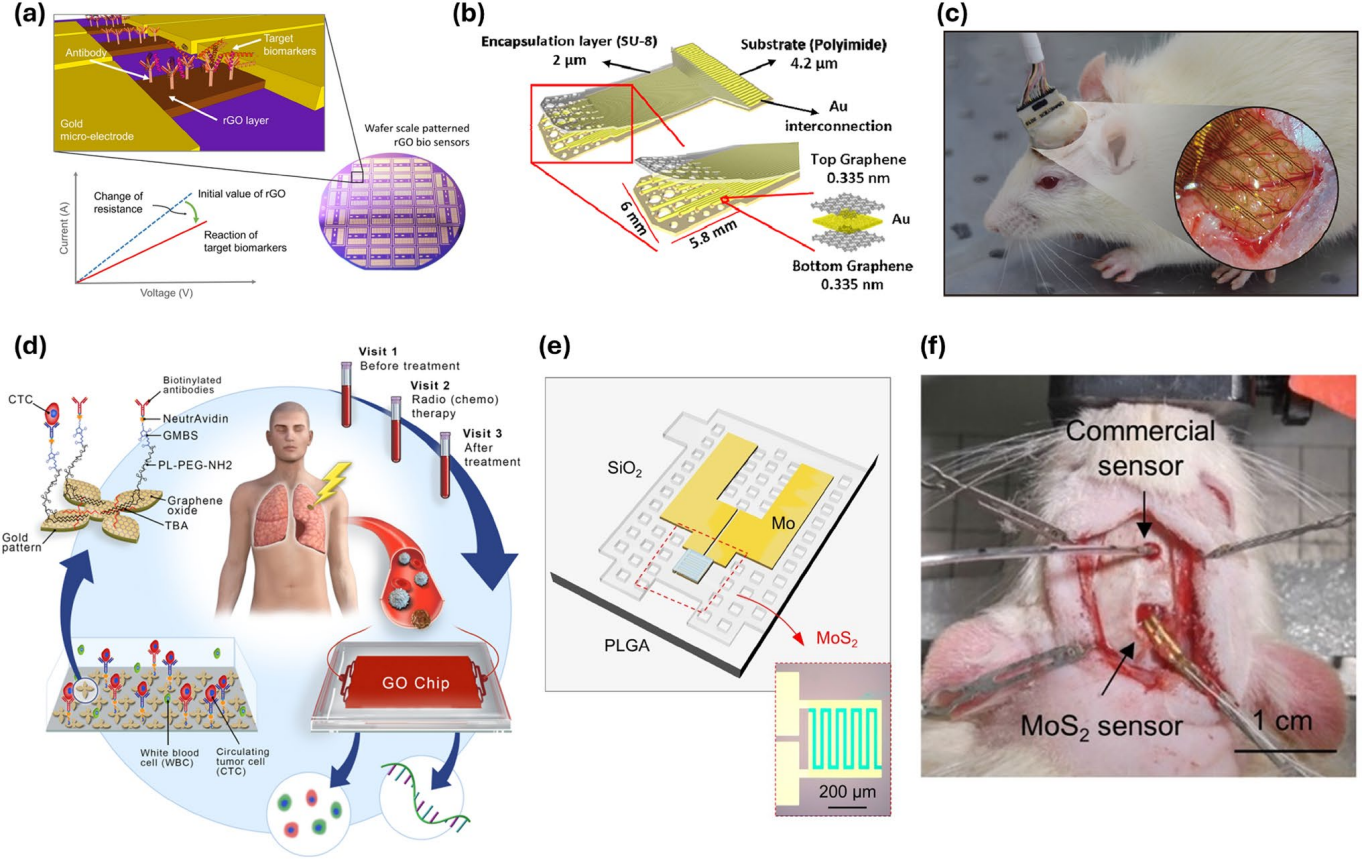
**a** Schematic of the large area wafer-scale fabricated rGO biosensors with immobilized antibody. Reproduced with permission [146]. Copyright (2016), Springer Nature. **b** Device structure of graphene (Gr/Au/Gr) electrode. **c** Photograph of rat with designed a hybrid graphene electrode. Panels **b** and **c** are reproduced with permission [151]. Copyright (2023), Springer Nature. **d** Schematic of GO chip configuration and work mechanism of sample collection and circulating tumor cell isolation. Reproduced with permission [148]. Copyright (2019), Springer Nature. **e** Structure of a MoS_2_-based biodegradable sensor. **f** Photograph of a MoS_2_-based bioabsorbable sensor implanted in a rat together with a commercial one. Panels **e** and **f** are reproduced with permission [150]. Copyright (2018), Springer Nature

2D materials have widely gained attention in biomedical applications because of their lightweight, high flexibility, good electrical properties, and excellent biocompatibility. Many innovations in materials and device manufacturing, and novel circuit design using 2D materials have offered notable progress in disease diagnosis and health monitoring [143]. Moreover, inevitable defects generated during the growth process of the 2D materials offer stronger interaction with biological targets compared to pristine adsorption sites. That would help to develop faster commercial sensors of the 2D materials by overcoming the growth challenge of high-quality 2D materials. So, extensive research efforts have been made to detect physiological information using 2D materials-based devices. Basically, physiological information is received from the human in terms of physical signals (temperature, electrocardiogram, electroencephalogram, and limb motions) and chemical signals (saliva, breathing biomarkers, plasma, and sweat). The 2D materials especially graphene and TMDs based biosensors have shown potential for non-invasive, continuous monitoring and identification of different health indicators. Since the first bio-device of graphene in 2008 with a resolution up to single-bacterium [144] different forms of graphene such as graphene oxide, and reduced graphene oxide have been explored in biomedical applications. The hygroscopic nature of the graphene oxide is suitable to detect water molecules during the change of humidity, and humidity sensors are utilized in various non-invasive applications including monitoring sweat rate during exercise and detecting rate of changes in moisture levels of respiratory infections. Lipani et al. reported a non-invasive approach for real-time monitoring of glucose using CVD-grown graphene [145]. The system measured the selective glucose level in the interstitial fluid via electroosmotic extraction through preferential hair follicular pathways. This non-invasive approach is more convenient for glucose detection than traditional finger-stick testing. Kim et al. reported a sensor using a wafer-scale rGO patterning film for low-concentration biomarker detection from plasma. The antibody-modified biomarker sensor changes the resistance value of the device after binding antibodies to the target biomarker [146]. The uniform GO deposited sensor chip and change in the resistance value of the device after biomarker detection are shown in Fig. 13a. The low-level detection of the biomarker in plasma was suitable for Alzheimer’s disease diagnosis. In addition, 2D materials are also utilized for the development of self-powered biosensors because of the low power consumption of 2D material devices. Li et al. reported a self-powered pH sensor using a 2D MoS_2_ and that sensor was driven by MoSe_2_-based piezoelectric nanogenerator [147]. The sensor exhibited high sensing performance with low response time (˃ 10 s) because of excellent electrical properties and the high surface-to-volume ratio of the 2D materials. In addition, microfluidic chips based on 2D materials are also popular for biosensing because of their high throughput and low cost. Wang et al. fabricated a microfluidic chip using graphene oxide for collecting whole blood from non-metastatic non-small cell lung cancer patients before, during, and after radiation or chemoradiation to monitor the effect of radiation chemotherapy on the PD-L1 expression, as shown in Fig. 13d [148].

Besides the development of wearable devices or non-invasive devices, they are also utilized in implantable devices because they have good stability and compatibility with biofluids and biological tissues [149, 150]. Conventional implantable devices create some common problems in terms of damage to the tissue, scars, or inflammation because of the rigid, bulky, and toxic nature of the used material. So, biocompatibility is most important for the implantable devices. These devices are basically utilized for monitoring electrophysiological signals and stimulating muscles and nerves. Lim et al. reported a hybrid graphene electrode for the diagnosis and treatment of epilepsy [151]. They fabricated a high-density, conformal flexible electrode array using graphene/Au/graphene with low impedance for brain activities signal, as shown in Fig. 13b. The graphene electrode array was mounted on the cortex of the rat (Fig. 13c) and information was received from different cortical sites and, neuro-stimulated for epilepsy treatment in free-moving rats. Chen et al. reported a MoS_2_-based bioabsorbable and multi-functional sensor for intracranial monitoring of pressure, temperature, strain, and motion in animal models (Fig. 13e, f) [150]. The CVD-grown monolayer MoS_2_ showed bio absorption through hydrolysis in aqueous solutions and long-term cytotoxicity and immunological biocompatibility in biofluids and tissues of live animal models. This sensor technology using 2D MoS_2_ provides clinically relevant roles in both diagnostic and therapeutic functions during the recovery process from traumatic brain injury.

### Artificial Intelligence

Computation and storage functionalities onto a single unit is one of the significant bottlenecks of the conventional von Neumann architecture because of the physical separation of logic and memory. The data transfer between logic and memory has slowed the computation power of the advanced Si processor. Significant computing power requirements for the operation of emerging artificial intelligence (AI) algorithms force to invention of alternating computing methods. On the other hand, size scaling and high energy demand of the Si-based CMOS technology have been triggered to search for new alternating computing processes. Inspired by the human brain’s biological neural networks, neuromorphic computing has captured significant attention to perform complex computation and big-data tasks with high energy efficiency. Basically, a neuromorphic computing system emulates the complex chemical behavior of synapses and neurons of the human brain at the device level. The success of several neuromorphic chips including TrueNorth, Loihi, and SpiNNaker has triggered the interest of the microelectronic industry in neuromorphic computing for AI [152] [153, 154]. Moreover, integration of the sensor with these neuromorphic computing platforms would be a crucial step to developing hardware-based AI.

**Fig. 14:**
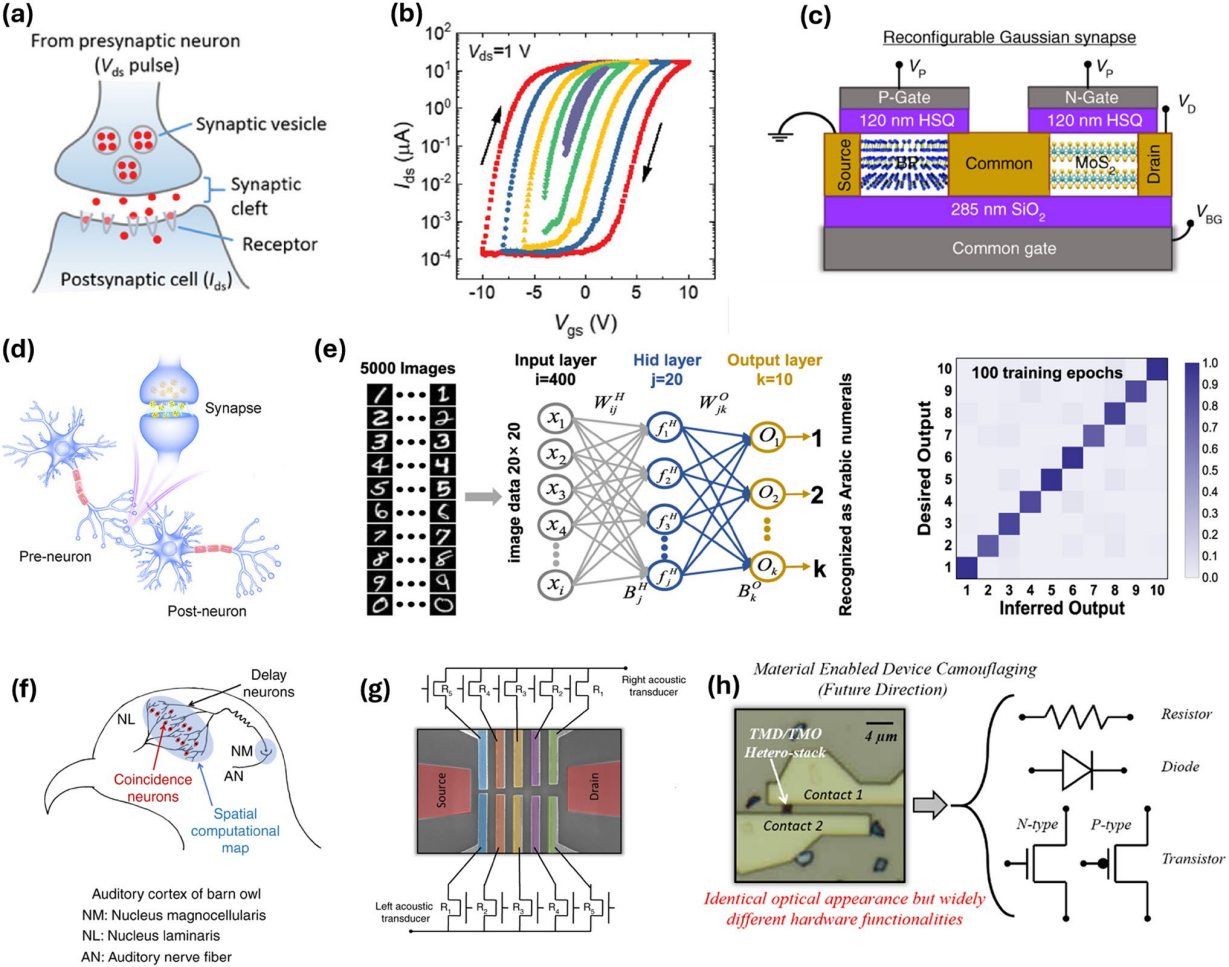
**a** Schematic of artificial synapse using 2D MoS_2_. **b** Transfer characteristics show increased hysteresis with increased gate voltage of the MoS_2_ based synapse. Panels **a** and **b** are reproduced with permission [156]. Copyright (2019), Wiley–VCH. **c** Schematic of Gaussian synapse using a dual gate of BP and MoS_2_. Reproduced with permission [157]. Copyright (2019), Springer Nature. **d** Schematic of a biological synapse. Reproduced with permission [158]. Copyright (2021), American Chemical Society. **e** Schematic of three-layer neuromorphic network for handwritten digit recognition, confusion matrix, and recognition rate as a function of training epoch for image training and testing. Reproduced with permission [159]. Copyright (2020), Springer Nature. **f** Schematic representation of auditory cortex barn owl. **g** Jeffress model for sound localization. Panels **f** and **g** are reproduced with permission [160]. Copyright (2019), Springer Nature. **h** An optical image of camouflaged TMO/TMD heterostructure can be either a resistor, a diode, or a transistor. Reproduced with permission [161]. Copyright (2021), American Chemical Society

In the last few years, 2D materials have emerged as potential candidates for neuromorphic computing device applications. Their inherent atom-level thickness, surface without dangling bonds, and remarkable electronic and optoelectronic properties make them suitable for next-level device scaling and bioinspired computing device applications. Moreover, the excellent flexibility, transparency, and printable nature of the many 2D materials are helpful for developing emerging advanced wearable devices. Many research efforts have been made to mimic biological computation at the device level using two (memristor) and three (memtransistors) terminal device structures. Various types of switching principles including resistive, phase change, ferroelectric, and atomic have been utilized for simulating synaptic plasticity. Artificial neural networks (ANN) algorithms mainly train the programmable conductance/resistance of the memristor, which is analogous to variation in synaptic weight, and further hardware extension leads to different neuromorphic computing applications [155]. Despite the simple device structure, easy mechanism, and high-density integration, these memristors have some issues such as destructive read operations, complex neuron mimicry, and inherent stochasticity. In a three-terminal device structure (transistor), the third terminal (gate) as a presynaptic node and source/drain as the postsynaptic node are mainly exploited in various devices such as floating gate memory, gate-tunable memory, and memtransistors for realizing artificial synaptic functionalities. Wang et al. realize an artificial synapse using three terminals transistors of monolayer polycrystalline-MoS_2_ (Fig. 14a) [156]. The device emulates biological synapse behavior by applying presynaptic input on either a drain or gate terminal and that is highly helpful for developing complex artificial brain-like biological structures. Moreover, the gate tunability function provides excellent cycling endurance with an operation of nonvolatile memory functions. The huge hysteresis in the transfer curve of the polycrystalline MoS_2_ transistor enables it for synapse applications. The charge trapping/de-trapping in trap states of the polycrystalline MoS_2_ was responsible for this huge hysteresis during the gate voltage scan (Fig. 14b). Besides the ANN, probabilistic neural networks (PNNs) are also utilized to mimic the fundamental biological functions of the brain. Although PNN is inspired by mathematical algorithms rather than neural biological functions. Sebastian et al. demonstrated a Gaussian synapse for the realization of the PNN using heterostructures of dual-gated MoS_2_ and black phosphorus (BP) FETs, as shown in Fig. 14c [157]. The Gaussian synapse classified brainwave patterns using probabilistic neural networks. The threshold engineering in dual-gated MoS_2_ and BP FETs is exploited for dynamic modulation of the amplitude, mean, and standard deviation of the Gaussian synapse. Furthermore, Liu et al. demonstrate an optoelectronic artificial synapse with stable bipolar resistive switching using titanium trisulfide (TiS_3_) (Fig. 14d) [158]. These types of artificial optical synapses have been utilized in advanced neuromorphic vision systems. In addition, Wang et al. demonstrated multiply-accumulate operations using in-memory computing architecture of 2D MoS_2_-based two-transistor-one-capacitor (2 T-1C) configuration [159]. In this work, three layers fully connected neural network was constructed of input and output neurons with 20 hidden layers of neurons for handwritten digit recognition, as shown in Fig. 14e. Ex-situ trained neural network based on experimental results exhibited excellent accuracy of 90.3% for image recognition. Das et al. reported a biomimetic transistor of 2D MoS_2_ for audiomorphic computing [160]. They used the Jeffress model of sound localization to mimic the auditory cortex of a barn owl and the device consists of multiple split-gates with nanogaps and tunable RC circuits for imitating the spatial map of coincidence detector neurons and for imitating the interaural time delay neurons, respectively, as shown in Fig. 14f, g. On the other hand, hardware security is also a challenge for emerging IoT systems. Reverse engineering (RE) is one of the main hardware security threats to the IC and is suitable for extracting device-level functionalities for identifying the device technology. IC camouflaging is an emerging significant obfuscation method to hide the IC functionality. In this context, Wali et al. utilized the heterostructure of TMD and its oxide TMO to hide the functionality of the device as well as circuits (Fig. 14h) [161]. Logic gates based on these heterostructures exhibited resilience to satisfiability solver and automatic test generation patterns attacks.

### Quantum Technologies

**Fig. 15:**
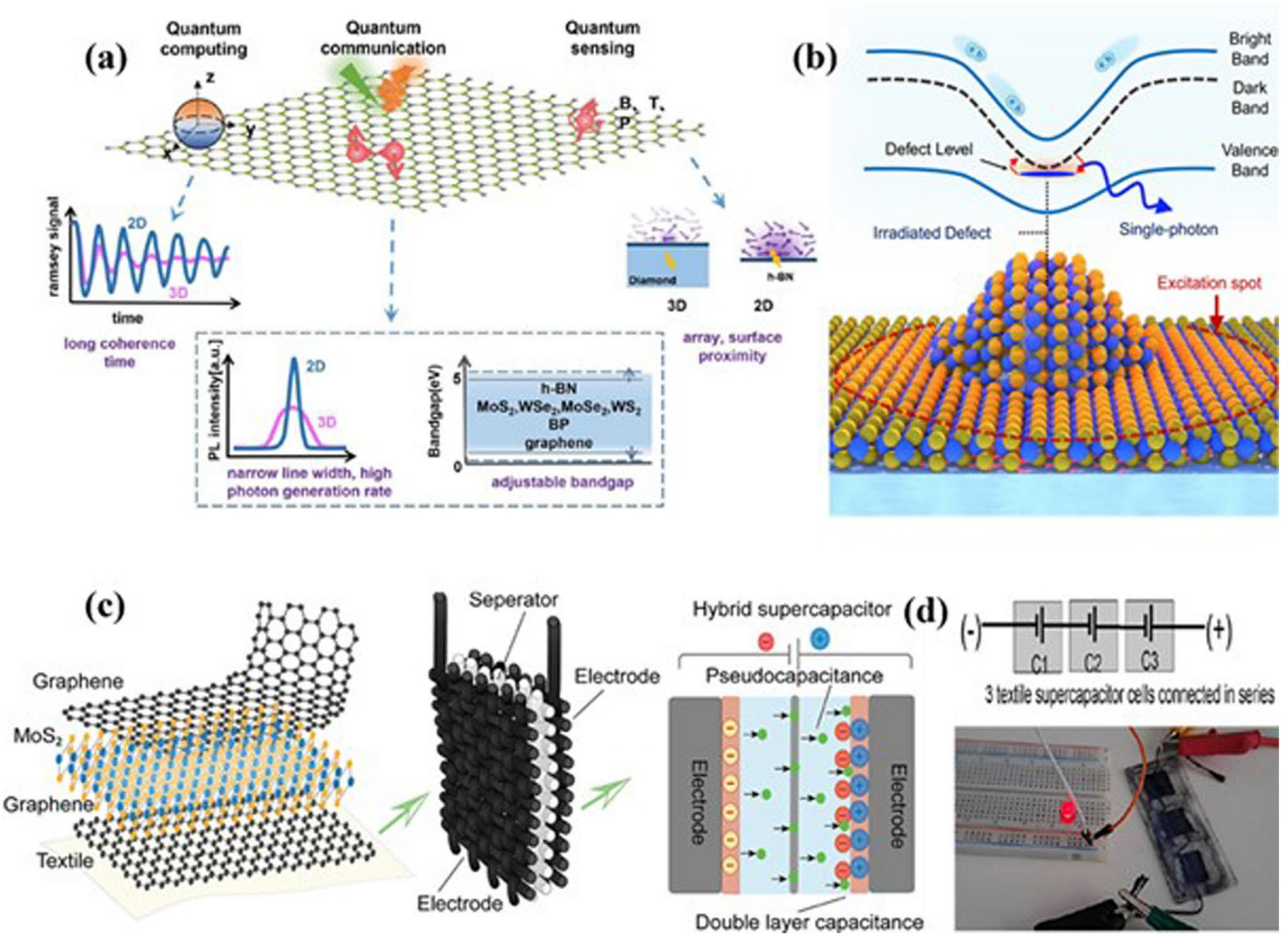
**a** Schematic represents the advantages and applications of the 2D materials in quantum technology. Reproduced with permission [163]. Copyright (2024), Springer Nature. **b** Schematic illustration of strain and defect engineered WSe_2_ based single-photon emitter. Reproduced with permission [167]. Copyright (2020), Springer Nature. **c** Schematic of e-textile supercapacitor using heterostructure of graphene and MoS_2_. **d** Image of powering LED using three capacitors in series. Panels **c** and **d** are Reproduced with permission [170]. Copyright (2023), American Chemical Society

In the past few years, quantum technology has been at the forefront of state-of-art research and innovation in different fields including computing, communication, sensing, and storage because of its unique advantages over classical technology. Basically, spin state superposition, entanglement, and coherence effects drive the quantum technology. In this context, 2D materials and their van-der-Waals heterostructures with excellent quantum phenomena are promising candidates for the development of various future quantum technologies [162]. The utilization and advantage of the 2D materials in different quantum applications including computing, communication, and sensing are shown in Fig. 15a [163]. Besides enabling artificial states of quantum matter, the 2D material systems show also huge potential on solid-state platforms for quantum technology applications [164]. Moreover, 2D materials-based vdW heterostructures after stacking 2D materials with various sequences, different layer spacing, and changing the relative angle between material layers lead to generating new quantum effects. In regards to quantum computing, qubits as a fundamental unit of information have been realized in various forms such as quantum dot qubits, defect spin qubits, superconducting qubits, and topological qubits using the 2D materials systems because of their peculiar band structures and unique quantum phenomena. The 2D TMDs materials exhibit faster qubit operations than the graphene because of their strong intrinsic spin–orbit coupling and large exciton binding energies [165]. Further, the susceptibility of defect spin qubits to the environment can be overcome using the 2D vdW heterostructures. Moreover, vertical stacking of the 2D materials (2D materials having superconductivity and insulation behavior) provide a clean interface and that help to develop Josephson junctions on the superconducting qubits platform. The van-der-Waals heterostructures are promising for topological quantum computing and topologically qubits are better protected against disorder [166]. In the context of quantum emission for quantum communication applications, 2D materials have been explored in different quantum emission phenomena including single photon, biexciton, and valley exciton. Parto et al. controlled the exciton characteristics of the 2D WSe_2_ using the defect and strain engineering for single-photon emitters at 150 K [167]. Figure 15b illustrates the site-controlled single-photon emitters in 2D WSe_2_. The 2D TMDs materials exhibit single-photon emission at low temperatures, while h-BN shows emission even at room temperature because of its strong exciton binding. On the other hand, discrete and tunable energy levels of the 2D materials help to exploit the properties of quantum states for quantum sensing applications. The negatively charged boron vacancies in the 2D h-BN support to sense of pressure, strain, and temperature because these vacancies exhibit good quantum coherence and single-spin addressability [168, 169]. In short, quantum technology applications using 2D materials are in a nascent stage, so they require continuous exploration.

### Energy Storage Devices

Depletion of fossil fuel resources and their adverse impact on the environment emphasize to use of clean and sustainable energy sources and storage devices. The intermittent behavior of renewable energy resources including solar, and wind increases the importance of suitable energy storage devices such as supercapacitors and batteries. Because these energy resources are highly dependent on the location, weather, and individuals. Moreover, emerging electric vehicles and consumer electronics devices have also demanded uninterrupted power supply. From this view, energy storage devices with high power and current densities are needed to address these energy crises. In the past few years, 2D materials such as graphene, black phosphorus, MXenes, and TMDs have gained significant interest in the development of energy storage devices [171, 172]. Their unique proprieties including atomically thin layered structure, high effective surface area, and excellent electrochemical properties make them promising materials in the energy storage field. Moreover, layered structures are highly stable during charging and discharging cycles and render additional sites for ions in energy storage. The metal electrodes play a vital role in achieving high performance in storage technologies such as electrochemical capacitors and metal-ion batteries. Graphene with excellent conductivity, good functionality, and more importantly the highest surface area of 2630 m^2^ g^−1^ among all carbon materials is an ideal electrode for energy storage devices. In the past few years, 2D MXenes and their composites have garnered huge interest in the development of energy storage devices because of their exceptional electrochemical and electrical properties. A supercapacitor is made from two symmetric electrodes separated by a membrane, and both electrodes are connected through ions of electrolytes. These electrochemical capacitors as supercapacitors store energy in two forms either electrochemical double layer (EDL) (electrolyte ions collected at the electrode/electrolyte interface) or through Faradaic redox reactions (involvement of the electrode material’s surface regions), known as pseudocapacitance [173]. From this view, materials should have both properties for efficient and robust operation of the energy storage device. However, it is difficult to find pseudocapacitance and EDL properties in a single energy storage material. Nevertheless, energy storage by ion adsorption at the electrode/electrolyte interface and additional contribution from Faradaic redox reactions can be achieved by the synthesis of 2D materials-based heterostructures. Islam et al. reported a wearable textile supercapacitor using the heterostructures of graphene and MoS_2_ [170]. They used a controllable microfluidization technique for highly scalable wearable e-textiles manufacturing, as shown in Fig. 15c. The supercapacitor showed a very high performance as graphene possesses an intrinsically high electrical conductivity and MoS_2_ exhibits a tuneable bandgap. An aerial capacitance of ∼105.08 mF cm^–2^, power density of ∼1604.274 μW cm^–2^, and energy density of ∼58.377 μWh cm^–2^ was obtained by using this supercapacitor. The heterostructure textile-based supercapacitor combined the working mechanism of pseudocapacitance and double-layer capacitance and hence acts as a hybrid capacitor. Further, three capacitors connected in series glowed LED and that confirms the practical applicability of the device, as shown in Fig. 15d. Yun et al. reported a flexible wire-shaped supercapacitor and a layer-by-layer assembly process was used for fabricating a wire-shaped supercapacitor on conducting carbon yarn using rGO and Ti_3_C_2_T_*x*_ MXene [174]. The supercapacitors showed high areal capacitance (40.8 mF cm^−2^), volumetric capacitance (2193 F cm^−3^), and specific capacitance (237 F g^−1^) and exhibited excellent mechanical stability with good retention ability of 90% after 200 bending cycles. On the other hand, batteries are widely exploited as main energy storage devices for consumable electronics and emerging electric vehicles. The 2D materials have been explored in different types of batteries including lithium-ion batteries, sodium-ion batteries, and magnesium-ion batteries, etc. Hwang et al. reported an anode electrode for lithium-ion batteries using the disordered graphene-like structure of the MoS_2_ nanoplates [175]. The high Li-ion storage capacity and good electrochemical performance were shown because of the larger interlayer distance of the MoS_2_ nanoplates and disordered graphene-like morphology. Furthermore, MXenes have much explored and shown huge potential to fabricate anode electrodes for Li-ion batteries. In the context of sodium-ion batteries, layered 2D TMDs, and MXenes materials are compatible with anode electrodes because graphite electrodes could not be suitable for the sodium-ion batteries owing to the large size of the Na ion [176].

### Sensors

**Fig. 16:**
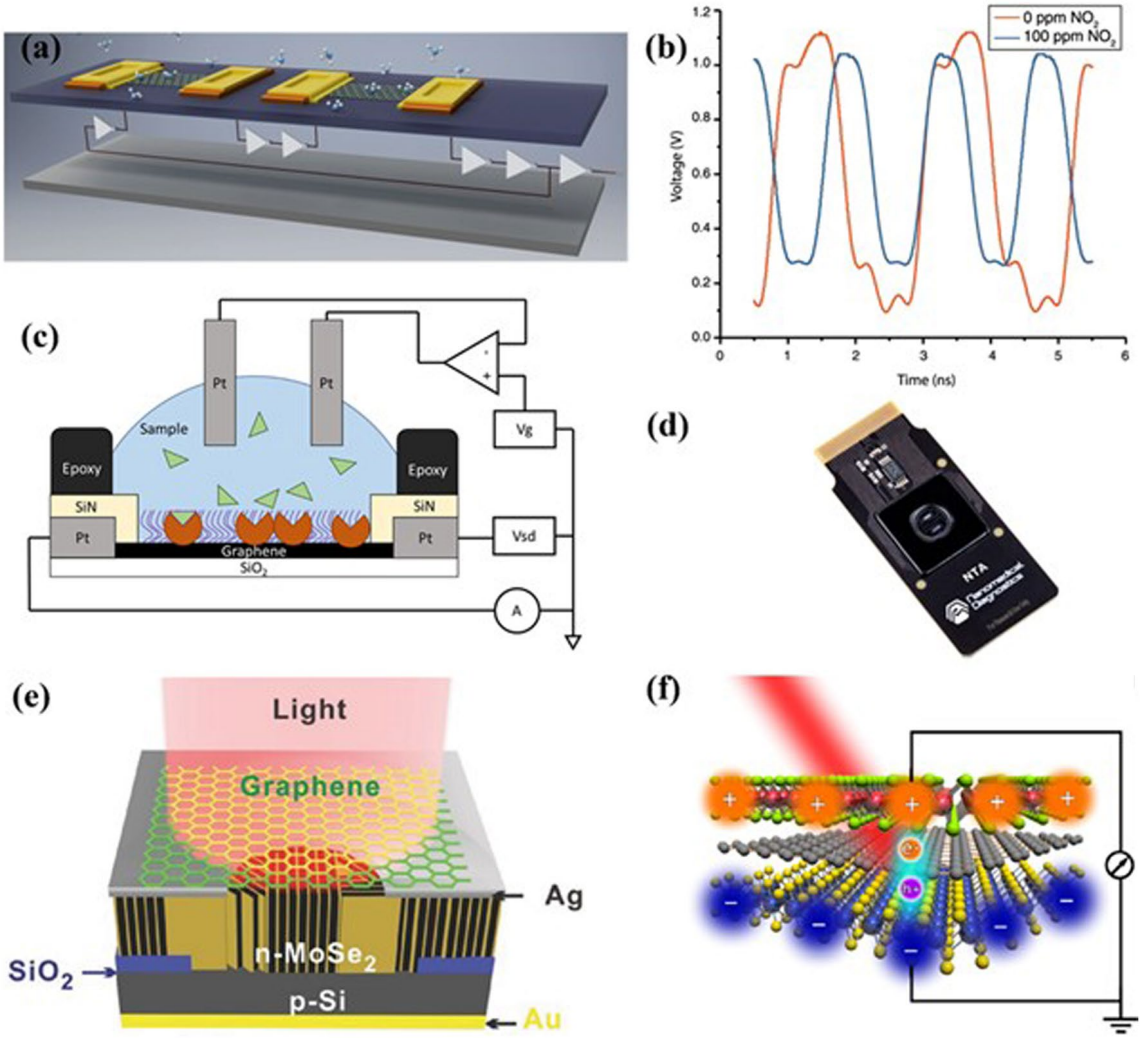
**a** Graphene chemiresistor gas sensors are integrated with commercial Si transistor chips to form a ring oscillator circuit. **b** Changes in frequency and phase to a varied NO_2_ gas concentration. Panels **a** and **b** are reproduced with permission [179]. Copyright (2017), Springer Nature. **c** Device structure and read-out circuit, and **d** a complete device image, of the commercial graphene-based biosensor. Panels **c** and **d** are reproduced with permission [180]. Copyright (2018), Springer Nature. **e** The Gr/MoSe_2_/Si photodetector with graphene transparent electrode. Reproduced with permission [184]. Copyright (2016), Wiley–VCH. **f** Broadband photovoltaic detector of p–g–n heterostructure (MoS_2_ –graphene –WSe_2_). Reproduced with permission [185]. Copyright (2016), American Chemical Society

Sensors play a crucial role in the development of the current IoT ecosystem. The sensor detects physical or chemical stimuli from the surrounding environment and converts them to an electrical signal. From this view, the inherent property of high surface-to-volume of the atomically thick 2D materials provides a large specific surface area for more interaction with external surrounding stimuli compared to other structural materials. External gas, chemical vapor, biomolecules, humidity, and temperature generate electronic perturbations on the surface of the 2D materials. Effective electronic perturbation plays a significant role in the development of highly sensitive and fast sensor devices. Gas, chemical, and biological molecules interact on the surface of the 2D materials through physisorption (vdW interaction) and chemisorption (interaction on defect, vacancy on the surface of the 2D material) [177]. That electronic interaction gives rise to a charge transfer gas sensing mechanism. Single gas (NO_2_) molecule detection at room temperature by graphene sensor in 2007 has provided interest in exploring the 2D materials in the sensing field [30]. Many 2D materials have shown potential for real-time detecting different gases (NO_2_, NH_3_, SO_2_, CO_2_, etc.), volatile organic compounds (VOCs), and biomolecules at room temperature. Detection of the gas at room temperature using 2D materials is a big achievement in the gas sensing field because commercial gas sensors based on metal oxide semiconductors detect the gas at elevated temperatures (˃ 150 °C). However, incomplete recovery at room temperature and susceptibility of the 2D materials toward the atmosphere is still a challenge for the 2D materials-based gas sensor. Several innovative strategies have been utilized to address these challenges. External optical or thermal energy is supplied to the sensor to achieve complete recovery because the provided energy above the threshold is sufficient to break strong interaction between gas molecules and adsorption sites [178]. Moreover, encapsulation of the 2D materials surface with other materials helps to improve the stability of the 2D materials. In addition, functionalization and hybridization of the 2D sensing layer with other nanomaterials (quantum dots, nanoparticles, nanowires) have further improved the sensing performance of the 2D materials-based gas sensors. The 2D material-based sensors have achieved significant progress in the gas sensing field and provide promise to employ them in advanced IoT and mobile applications. From a technological perspective, Zanjani et al. integrated graphene on the top of a commercial Si-CMOS chip (fabricated with 0.18 µm CMOS technology) and performed the sensing for NO_2_ and NH_3_ gases [179]. The monolithic CMOS-graphene gas sensor device is shown in Fig. 16a. A change in conductivity of the graphene upon exposure to gas influences the output signal of the readout circuit of the Si-CMOS chip. The decreased resistance value of the p-type graphene after electron extraction by NO_2_ reduced the propagation delay of a ring oscillator in the readout circuit and that results in increased corresponding output frequency, as shown in Fig. 16b. The monolithic CMOS-graphene gas sensor exhibited good sensitivity to NO_2_ and NH_3_ at atmospheric ambient. Furthermore, tuneable band gap, biocompatibility, and strong photoluminescence of the 2D materials support to detection of low concentrations target biological molecules from the complex biological samples. For biomolecule detection, field-effect transistor (FET) device structure is mostly used for 2D materials compared to capacitive and resistive biosensor structures. The electron transport of 2D material as the channel of the FET changes after the interaction of target biomolecules. Therefore, high-mobility materials are needed to detect the biomolecules. Moreover, surface functionalization/modification of the channel and novel structure design improve the selectivity of the biosensor toward specific analytes. Nowadays, graphene-enabled field-effect biosensors are commercially available for the detection of selective biomolecules (Fig. 16c) [180]. The performance of the biosensor is comparable (or superior) to conventional biosensor devices. The commercial biosensor chip is shown in Fig. 16d. In addition to change of electrical parameter such as current, resistance, and capacitance values of the 2D material-based electronic devices under the exposure the gases, modulation of optical properties of the optical devices in presence of the gases is also a promising approach for selective and highly sensitive gas detection. In this context, Yao and his group have integrated graphene with various optical devices for gas sensing applications. They have developed graphene heterogeneous D-shaped fiber device [181], graphene over-modal microresonator [182], and graphene-functionalized microlaser [183] for individual gas molecules detection and identification of gas molecules from the gas mixture. On the other hand, 2D materials have also been exploited for sensitive detection of pressure, temperature and humidity.

In gas sensing, 2D materials like graphene offer remarkable enhancements in sensitivity and detection limit through advanced control mechanisms such as working point adjustment, leveraging their unique electronic and optical properties. A significant breakthrough was demonstrated using graphene integrated into a bipolar-junction-transistor heterogeneous D-shaped fiber (GhDF), where ultrasensitive four-wave mixing (FWM) down-conversion enabled the detection of individual gas molecules in real time under vacuum conditions [181]. This was achieved through the steep change in FWM conversion efficiency as the graphene Fermi level (E_F_) approached 0.4 eV, a condition that can be finely tuned via external electrical controls. Gas adsorption modulates graphene's E_F_ either by charge transfer or impurity doping, significantly altering its optical and electronic behavior. For instance, NH_3_ acts as a donor molecule, contributing electrons and directly shifting E_F_, while CO_2_ introduces scattering impurities, changing graphene’s refractive index. Such mechanisms allow precise adjustments to the sensing working point, enhancing the detection of specific gases or broader chemical classes. For NH_3_, the detection relies on lone electron pairs of nitrogen, enabling the sensing of related amino molecules like NH_2_CH_3_ or N(CH_2_CH_3_)_3_. The combination of graphene's strong nonlinearity, electrical tunability, and integration with photonic platforms enables high-sensitivity detection that was previously unachievable in integrated photonic devices.

Moreover, integrating 2D materials with advanced optical technologies, such as optical frequency combs and microcavity mode splitting, enables real-time multi-component gas detection with unprecedented single-molecule precision. Optical microcavities are powerful tools for enhancing light–matter interactions, crucial for precise sensing applications. However, the intrinsic inertness of pristine microresonators limits their potential in emerging fields, such as gas detection. Functionalizing these microcavities with atomically thin materials, such as graphene, overcomes these limitations and introduces novel sensing capabilities. Graphene’s exceptional optical and electronic properties, including strong nonlinearity, tunability, and high sensitivity to adsorption, make it an ideal material for integrating with optical systems [183]. For instance, functionalized microcavity sensors, like erbium-doped microspheres with graphene layers, generate laser lines in distinct mode families using a single pump source. These co-generated modes interfere, producing beat notes in the electrical domain with sub-kHz accuracy, thanks to graphene-induced intracavity backward scattering. This allows precise identification of multiple gas species in mixtures, even without a laboratory setup. Similarly, graphene-functionalized soliton frequency combs have been shown to enable real-time single-molecule detection through the unique properties of Stokes solitons [182]. By asymmetrically depositing graphene on microresonators, spectrally trapped solitons with locked repetition rates but distinct offsets are generated in the same device. These solitons produce highly sensitive beat notes in the electrical domain, with their stability allowing tracking of frequency shifts with uncertainty below 0.2 Hz. Such sensitivity enables the detection of individual gas molecules, as demonstrated with NH_3_ adsorption events, where graphene's interaction with gas molecules directly modulates the Fermi level (E_F_), leading to measurable optical responses. Furthermore, this method enables the detection of multi-component gas mixtures by analyzing the beat note responses of different Stokes solitons to specific gases, achieving high selectivity and quantitative accuracy. The hybridization of microcavities with 2D materials thus combines soliton stability, electrical tunability, and ultrasensitive light-matter interactions, enabling real-time detection of individual molecules and gas mixtures. These advances pave the way for compact, high-performance optical sensors with applications in environmental monitoring, healthcare diagnostics, and industrial safety.

### Photodetectors

The photodetector is a famous optoelectronic device that plays a crucial role in different applications including medical imaging, optical sensing, and optical communication. In the past few years, 2D materials have attracted tremendous attention in the development of photodetectors because of their excellent optical, and electrical properties and flexible integration. The 2D materials have been utilized in effective photodetection for an ultrabroad wavelength range from ultraviolet to THz [186]. In the context of 2D materials photodetection, the incident photons on the active region of the device lead to an electrical signal through different photo-sensing mechanisms including photovoltaic, photoconductive, photo-gating, photo-thermoelectric, bolometric, direct and Fowler–Nordheim tunnelling. Despite the different photodetection mechanisms, conventional figures-of-merit of the photodetector such as photoresponsivity, quantum efficiency, detectivity, response time, optical bandwidth, etc. are utilized to evaluate the performance of the 2D materials-based photodetectors. The working of 2D material photodetectors based on photoconductive effect are widely explored and that results in high dark current and low detectivity. The gate modulation in phototransistors helps to improve the sensitivity and reduce dark current. Moreover, utilization of the ferroelectric materials in gate dielectric also supports enhanced sensitivity through reducing dark current by exploiting the local electrostatic field of the ferroelectric material. Schranghamer et al. reported an ultra-scaled and low-power phototransistor using the 2D MoS_2_ with an active area of device 0.0065 µm^2^ [187]_._ The device showed high responsitivity of 8.84 × 10^8^ A W^−1^ and detectivity of 1.65 × 10^13^ Jones by exploiting the photogating effect through trapping of photogenerated holes in high k dielectric Al_2_O_3_ under depletion mode operation.

The individual 2D material-based device shows low absorption of light because of its inherent atomically thin thickness. A monolayer 2D material-based photodetector exhibits lower photoresponsivity compared to conventional commercial photodetectors. The graphene provides a wide spectral range owing to its zero-band gap, but the short lifetime of photogenerated carriers impedes large photocurrent. In contrast, several 2D TMDs materials have large band gaps and large carrier lifetimes. From this view, the construction of vdW heterostructures using 2D materials is a promising method to exploit their synergistic effects in photodetection. Yu et al. reported a broadband and self-powered photodetector using wafer-scale PtTe_2_/graphene heterostructures [188]. The photodetector exhibited good photodetection performance in broadband wavelength range from 405 to 1850 nm. The photodiode showed high D* (~ 2.58 × 10^10^ Jones) and a fast response time of ∼8.4 μs. Long et al. inserted graphene in between the p–n vdW heterostructure of MoS_2_/WSe_2_ and formed a MoS_2_ –graphene –WSe_2_ heterostructure-based photodetector, as shown in Fig. 16f [185]. Graphene with zero energy band gap helps to provide a wide spectral range for effective light absorption. The photodetector showed good photodetection in the broadband range from visible to short-wavelength infrared range and exhibited specific detectivity of up to 10^11^ Jones in the near-infrared region at room temperature. A vertical device structure of graphene/MoSe_2_/Si heterostructure exhibited excellent photoresponse in a wide range of 350–1310 nm and importantly, an ultrafast photoresponse speed of ≈270 ns [184]. The photodetector device is shown in Fig. 16e. The transparent top graphene electrode of the device enhances the carrier collection and reduces the recombination at electrode junction because of the strong built-in electric field in graphene/MoSe_2_/Si heterojunctions. Moreover, transparent electrodes improve the photoresponse by avoiding the shading of the active area of the device compared to conventional metal electrodes. Among the different vdW heterostructures of 2D materials, the integration of 2D materials with Si has grabbed more attention in the development of advanced photodetector devices. The matured silicon photonics offer low-cost easy read-out circuits platforms and are compatible with conventional CMOS technology.

## Concluding Remarks and Outlook

The increasing demand of flexible, wearable, and portable electronics have aroused worldwide interest in atomically thin 2D materials. Over the past decade, the advent of newly discovered properties of 2D materials have transformed the smart devices, ranging from flexible electronics to quantum technologies. As conventional materials undergo a transition from 3 to 2D by reducing their thickness at atomic levels in one direction, some exciting physics occurs particularly due to quantum confinement. The occurrence of quantum physics makes the 2D materials more sensitive to environmental conditions and external stimuli. From a smart device perspective, this gives an extra edge for determining different parameters precisely. For instance, the presence of a single atom of hazardous gas molecule could be detected by 2D materials. Despite the promising applications of 2D materials in developing smart devices, we still face many challenges for their practical implementation. For further development of 2D-based smart devices, the following aspects need to be addressed:

1. Several commercial enterprises have already started making smart devices using 2D materials, particularly graphene. However, the cost is relatively high due to the complexity associated with large-scale production of 2D materials. Moreover, the air stability of some of the 2D materials makes them incompatible for developing smart devices. For instance, phosphorene is unstable under ambient conditions as it would get easily oxidized. Hence, some complex techniques such as chemical functionalization, elemental doping, and oxidation-resistant coatings have been adopted to improve the air stability of 2D-based smart devices.
2. Mixed dimensional heterostructures created by combining different dimensional materials offers unique device functionalities, however, synthesizing defect-free and stable interfaces remains a significant challenge. The device performance further deteriorates with increasing differences between thermal expansion coefficients and lattice parameters of the constituent materials due to mechanical strain.
3. Another difficulty in developing mixed-dimensional heterostructures-based smart devices is making reliable contact with an individual material. Contact resistance for short-channel devices is another crucial aspect that requires immediate attention. Contact engineering is required at the metal-semiconductor interface to make efficient charge transport across the heterointerfaces. By using some solvent-based techniques or dry chemical processes, we can achieve the optimal contacts for enhancing the overall performance of the sensors.
4. The interfacial physics specifically for 2D-based heterojunctions is still elusive. For instance, the formation of the depletion layer between atomically thin materials requires further study as the order of depletion width may be much higher than the physical thickness of these materials. Therefore, interfacial dynamics at mixed-dimensional heterojunctions need to be explored to understand the carrier density distribution and their transport processes. Moreover, some of the newly discovered 2D materials such as phosphorene, silicene, and arsenene have a very short history. Hence, the inherent properties of these materials such as electronic, chemical, and mechanical properties need to be explored to unearth their full potential for developing smart devices.
5. Integrating 2D materials with existing Si technology is very difficult due to difference in device fabrication processes and architectures. This integration requires advanced growth, transfer, and encapsulation methodologies while carefully understanding the device physics and processing compatibility.

In summary, the incorporation of 2D materials for developing smart devices has a huge potential to bridge the technological gap between conventional and modern electronic devices. However, the device architecture, stability, performance improvement strategies, and other technical aspects need extensive studies to pave the way for next-generation smart devices. By addressing these challenges, 2D materials are poised to unlock new levels of performance and functionality in smart devices, potentially transforming fields ranging from flexible electronics to quantum computing and beyond.
